# Assessment of an Interactive Digital Health–Based Self-management Program to Reduce Hospitalizations Among Patients With Multiple Chronic Diseases: A Randomized Clinical Trial

**DOI:** 10.1001/jamanetworkopen.2021.40591

**Published:** 2021-12-28

**Authors:** Scott A. Lear, Monica Norena, Davina Banner, David G. T. Whitehurst, Sabrina Gill, Jane Burns, Damanpreet K. Kandola, Suzanne Johnston, Dan Horvat, Kaitey Vincent, Adeera Levin, Annemarie Kaan, Harriette G. C. Van Spall, Joel Singer

**Affiliations:** 1 Faculty of Health Sciences, Simon Fraser University, Vancouver, British Columbia, Canada; 2 Division of Cardiology, Providence Health Care, Vancouver, British Columbia, Canada; 3 Population Health Research Institute, McMaster University, Hamilton, Ontario, Canada; 4 Centre for Health Evaluation and Outcome Sciences, Vancouver, British Columbia, Canada; 5 School of Nursing, University of Northern British Columbia, Prince George, British Columbia, Canada; 6 Division of Endocrinology, University of British Columbia, Vancouver, British Columbia, Canada; 7 Department of Physical Therapy, University of British Columbia, Vancouver, British Columbia, Canada; 8 Department of Health Sciences, Brock University, St Catharines, Ontario, Canada; 9 Department of Family Practice, University of British Columbia, Vancouver, British Columbia, Canada; 10 Division of Nephrology, University of British Columbia, Vancouver, British Columbia, Canada; 11 School of Nursing, University of British Columbia, Vancouver, British Columbia, Canada; 12 Department of Medicine, McMaster University, Hamilton, Ontario, Canada; 13 School of Population and Public Health, University of British Columbia, Vancouver, British Columbia, Canada

## Abstract

**Importance:**

Digital health programs may have the potential to prevent hospitalizations among patients with chronic diseases by supporting patient self-management, symptom monitoring, and coordinated care.

**Objective:**

To compare the effect of an internet-based self-management and symptom monitoring program targeted to patients with 2 or more chronic diseases (internet chronic disease management [CDM]) with usual care on hospitalizations over a 2-year period.

**Design, Setting, and Participants:**

This single-blinded randomized clinical trial included patients with multiple chronic diseases from 71 primary care clinics in small urban and rural areas throughout British Columbia, Canada. Recruitment occurred between October 1, 2011, and March 23, 2015. A volunteer sample of 456 patients was screened for eligibility. Inclusion criteria included daily internet access, age older than 19 years, fluency in English, and the presence of 2 or more of the following 5 conditions: diabetes, heart failure, ischemic heart disease, chronic kidney disease, or chronic obstructive pulmonary disease. A total of 230 patients consented to participate and were randomized to receive either the internet CDM intervention (n = 117) or usual care (n = 113). One participant in the internet CDM group withdrew from the study after randomization, resulting in 229 participants for whom data on the primary outcome were available.

**Interventions:**

Internet-based self-management program using telephone nursing supports and integration within primary care compared with usual care over a 2-year period.

**Main Outcomes and Measures:**

The primary outcome was all-cause hospitalizations at 2 years. Secondary outcomes included hospital length of stay, quality of life, self-management, and social support. Additional outcomes included the number of participants with at least 1 hospitalization, the number of participants who experienced a composite outcome of all-cause hospitalization or death, the time to first hospitalization, and the number of in-hospital days.

**Results:**

Among 229 participants included in the analysis, the mean (SD) age was 70.5 (9.1) years, and 141 participants (61.6%) were male; data on race and ethnicity were not collected because there was no planned analysis of these variables. The internet CDM group had 25 fewer hospitalizations compared with the usual care group (56 hospitalizations vs 81 hospitalizations, respectively [30.9% reduction]; relative risk [RR], 0.68; 95% CI, 0.43-1.10; *P* = .12). The intervention group also had 229 fewer in-hospital days compared with the usual care group (282 days vs 511 days, respectively; RR, 0.52; 95% CI, 0.24-1.10; *P* = .09). Components of self-management and social support improved in the intervention group. Fewer participants in the internet CDM vs usual care group had at least 1 hospitalization (32 of 116 individuals [27.6%] vs 46 of 113 individuals [40.7%]; odds ratio [OR], 0.55; 95% CI, 0.31-0.96; *P* = .03) or experienced the composite outcome of all-cause hospitalization or death (37 of 116 individuals [31.9%] vs 51 of 113 individuals [45.1%]; OR, 0.57; 95% CI, 0.33-0.98; *P* = .04). Participants in the internet CDM group had a lower risk of time to first hospitalization (hazard ratio, 0.62; 95% CI, 0.39-0.97; *P* = .04) than those in the usual care group.

**Conclusions and Relevance:**

In this study, an internet-based self-management program did not result in a significant reduction in hospitalization. However, fewer participants in the intervention group were admitted to the hospital or experienced the composite outcome of all-cause hospitalization or death. These findings suggest the internet CDM program has the potential to augment primary care among patients with multiple chronic diseases.

**Trial Registration:**

ClinicalTrials.gov Identifier: NCT01342263

## Key Points

**Question:**

Does a digital health intervention that uses the internet to support patient self-management and self-monitoring and is implemented in primary care clinics reduce hospitalizations among patients with multiple chronic diseases?

**Findings:**

In this randomized clinical trial of 230 participants with multiple chronic diseases who were recruited from 71 primary care clinics. No significant difference in all-cause hospitalizations among participants who received the digital health intervention compared with usual care was found after 2 years; fewer participants in the intervention group were admitted to the hospital or experienced the composite outcome of all-cause hospitalization or death.

**Meaning:**

In this study, a digital health intervention did not reduce hospitalizations; however, the findings suggest a digital health intervention supporting patient self-management and self-monitoring has the potential to augment primary care among patients with multiple chronic diseases.

## Teaser

This randomized clinical trial assesses whether an internet-based self-management and symptom monitoring intervention reduces all-cause hospitalization among patients with multiple chronic diseases compared with usual care.

## Introduction

Chronic diseases are the leading cause of death and hospitalization worldwide.^1,2^ Many chronic diseases share risk factors, and the presence of 1 factor often increases the risk of another. As a result, more than one-half of adults have more than 1 chronic disease (referred to as multimorbidity^3^).^4,5,6^ Adults with more than 1 chronic disease present a complex challenge to health care systems and are at greater risk of rehospitalization.^7^ However, traditional management, consisting of reactive and disease-specific specialist-initiated strategies, often results in health care inefficiencies and conflicting care plans.^8,9,10^ Therefore, frameworks designed for a single chronic condition are inadequate for addressing multimorbidity.^11,12,13^

Treatment of patients with multimorbidity is primarily managed by primary care physicians (PCPs) in coordination with other health care professionals.^14,15^ Implicit in care is successful patient self-management,^16,17^ which includes self-monitoring of symptoms,^18^ maintenance of healthy lifestyle behaviors, and management of medications. Among patients with heart failure, an estimated 50% of hospitalizations could be avoided through effective patient self-management.^19^ However, self-management interventions among patients with multimorbidity have had limited benefits and are hindered by a lack of high-quality randomized clinical trials.^20,21^

Digital health technologies have the potential to facilitate patient-centered care by improving accessibility and supporting self-management.^22^ The ongoing COVID-19 pandemic has accelerated the need for robust telehealth programs.^23,24,25,26^ To date, studies of telehealth programs^27,28,29,30,31,32^ have been predominantly limited to heart failure, reporting that patient self-management, symptom monitoring, and alerts may be capable of improving quality of life and reducing hospitalizations. However, these studies have not included patients from small urban and rural areas, where hospitalization and mortality rates are higher.^33,34,35^ We evaluated an internet-based self-management program for chronic diseases (internet chronic disease management [CDM] intervention) that was implemented within primary care among patients with multimorbidity living in small urban and rural areas. We hypothesized that individuals participating in this program would have fewer hospitalizations over a 2-year period compared with individuals receiving usual care.

## Methods

In this single-blinded randomized clinical trial, participants were recruited from primary care clinics in small urban and rural areas throughout British Columbia, Canada. Small urban and rural areas were defined by excluding areas that had ambulatory care clinics related to the target diseases at the time of recruitment. This process resulted in the exclusion of all 4 British Columbia census metropolitan areas and 2 of the province’s census agglomerations (Penticton and Vernon).^36^ The trial protocol is available in Supplement 1. This study was approved by Simon Fraser University and relevant regional research ethics boards. All participants provided written informed consent. The study followed the Consolidated Standards of Reporting Trials (CONSORT) reporting guideline for randomized clinical trials.^37^

Potential PCPs were identified using the registry of the College of Physicians and Surgeons of British Columbia. Participating PCPs identified patients who had 2 or more of the following 5 conditions: diabetes, heart failure, ischemic heart disease, chronic kidney disease, or chronic obstructive pulmonary disease (COPD). These diseases were selected because they were the leading causes of hospitalization in Canada,^38^ shared common risk factors, and involved patient self-management for their care. Recruitment occurred between October 1, 2011, and March 23, 2015. Patients were mailed a letter from their PCP informing them of the study and directing them to contact the study coordinator (K.V.). Patients who had daily internet access, were older than 19 years, and were fluent in English were eligible for inclusion. Patients were excluded if they had substantial comorbidities (apart from the 5 targeted conditions) that may have interfered with effective care management or if they were unable to provide informed consent.

### Baseline Assessment

Participants received a baseline assessment comprising questions about demographic characteristics, self-management ability, and psychosocial measures. Data on medical history, medications received, and smoking status were extracted from patient medical records.

### Randomization

Participants were randomized on a 1:1 ratio to receive either usual care or the internet CDM program using variable block sizes. Randomization was generated using the PROC PLAN module in SAS software, version 9.4 (SAS Institute Inc), by a statistician not associated with the study. The randomization list was incorporated into an Oracle-based password-protected website (Oracle Corp), to which the randomization assistant (who was not involved in recruitment or participant assessments) logged in for randomization. The randomization assistant informed participants of their group assignment and asked them not to reveal their assignment to the study research coordinator to retain assessment blinding.

### Usual Care

Participants randomized to receive usual care were given educational information regarding chronic disease management and a list of internet-based resources. Other than outcome assessments at 12 and 24 months, there was no contact between study personnel and participants in the usual care group. No attempt was made to control the level of care received by participants in the usual care group, and they were free to seek any type of care they wished during the study.

### Intervention

Coordinated care between the participant, the participant’s PCP, and the nurse managing the internet CDM program was embedded within the 2-year intervention. The internet CDM program was designed by an advisory committee of clinical researchers, PCPs, specialist physicians, allied health care professionals, digital health care professionals (including S.A.L., S.G., J.B., K.V., A.L., and A.K.), and 3 patient members. This committee designed the intervention and implementation of the study. Patient members pilot tested and approved the final version.

The internet CDM intervention was managed by a full-time nurse during standard weekday hours. The nurse was supported by a dietitian and an exercise specialist. After randomization, participants’ PCPs were contacted by the nurse to inform them of their patient’s participation, discuss the patient’s action plans, and review the ways in which the internet CDM program could best support shared patient care.^39,40^ To ensure PCP engagement, the intervention was designed to align with existing provincial payment codes for patients with complex conditions and for telephone consultations between nurses and PCPs.

Each participant was provided with unique login details and received training for the use of the CDM website. The nurse conducted an introductory telephone call to discuss the participant’s condition, codevelop an action plan, and set targets for relevant biometric data.

Participants routinely completed a symptom report consisting of questions about disease-specific symptoms, biometric data (eg, weight, blood pressure level, and blood glucose level), and a free-text comment field. Symptom-related items began with a yes or no question. The assessment items were developed by the advisory committee based on clinical expertise, disease presentation, and patient experience (eTable 1 in Supplement 2). If the participant answered yes to a symptom question, a follow-up question was asked. For example, participants with heart failure were asked, “Did you wake up feeling more short of breath?” If the participant answered yes, a follow-up question was asked, such as, “Compared to yesterday, are your feelings of shortness of breath: much better, a little better, no change, a little worse, much worse?”

The alert algorithm was developed based on guidelines at the time of the study,^41,42,43,44^ the expertise of the advisory committee, and patient feedback (Box). In addition, alerts were generated if participants reported they had not met targets set for body weight, blood pressure level, and blood glucose level (eTable 2 in Supplement 2). Targets could be adjusted based on discussion between the nurse and participant. Alerts were emailed to the nurse, who called the participant within 1 business day. Possible actions after an alert included continued support of participant self-management, recommendation for follow-up with their PCP (in which case the nurse called the PCP to discuss the participant’s case), or referral to the nearest hospital.

Box. **Alert Algorithm**

1. Patient answers “much worse” to 1 or more questions
2. Patient answers “a little worse” to 2 or more questions
3. Patient answers “a little worse” to the same question on 2 consecutive occasions
4. Patient missed completing a symptom report for 3 consecutive scheduled times
5. Patient enters a comment in the free-text comment box during their answering of questions/data entry
6. Nurse has not reviewed patient data for 2 or more consecutive weeks

Participants were prompted by email to complete the symptom report daily for 2 weeks. If no alerts were generated during this period, the frequency was reduced to once per week. If an alert was generated, participants continued answering the symptom report daily until 1 week of no alerts passed. At any time, the nurse, in consultation with the participant, could override the symptom report frequency. Every 8 weeks, participants answered a lifestyle questionnaire regarding medication adherence, diet, physical activity, mood or the presence of depression, and smoking habits. Standardized thresholds for these questions were set and used to alert the nurse and participant for possible referral to a dietitian or exercise specialist or for the recommended use of a psychosocial support workbook. Participants had access to a public forum, graphical presentations of their biometric data overlaid with relevant alerts, their action plan, and external online resources.

### Primary Outcome

The primary outcome was the number of all-cause hospitalizations from the time of randomization to the end of 2 years. Data were collected at 1 and 2 years after randomization through a telephone interview and confirmed by hospital records.

Because prestudy data on hospitalizations in this population were limited, we chose a convenience sample of 300 participants and expected that 10% of participants would be unavailable for follow-up, resulting in a final sample of 270 participants. Preliminary data from our hospital-based clinic serving patients with multiple chronic diseases suggested a 25% reduction in hospitalizations compared with usual care over a 2-year period and a greater than 80% hospitalization rate for patients receiving usual care. With a hospitalization rate of 70% and a 25% reduction in hospitalizations due to the intervention, the statistical power was calculated at β = .78.^45,46^ However, recruitment ended at 230 participants because we had approached all potentially eligible PCPs in the province.

### Secondary and Exploratory Outcomes

Secondary outcomes consisted of hospital length of stay; quality of life using the Medical Outcomes Study 36-item Short Form survey, version 2 (score range, 0-100, with higher scores indicating less disability)^47^; self-management using the Health Education Impact Questionnaire (score range, 1-4, with higher scores indicating better self-management [with the exception of the emotional well-being dimension, for which higher scores indicate worse self-management])^48^; social support using the Medical Outcomes Study Social Support Scale (score range, 0-100, with higher scores indicating greater social support)^49^; and user login data. Exploratory and unregistered outcomes included the number of participants with at least 1 hospitalization, the number of participants who experienced a composite outcome of all-cause hospitalization or death, and the time to first hospitalization.

### Statistical Analysis

We used an intention-to-treat analysis. For the primary outcome, multivariable negative binomial regression analysis was used to assess the effect of the intervention relative to usual care. The model was adjusted for age, sex, and number of chronic conditions. We conducted a sensitivity analysis excluding participants who died during the study period because these participants could have contributed more hospitalizations to our outcome if they had not died.

A negative binomial multiple regression analysis was used to assess the effect of study group on the number of hospital days. Differences between the groups with regard to changes in quality of life, self-management, and social support were assessed using a 1-tailed Wilcoxon rank sum test. The outcomes of at least 1 hospitalization and the composite of at least 1 hospitalization or death from any cause were modeled using a multiple logistic regression analysis. We used Cox proportional hazards modeling to assess the effect of study group on the time to first hospitalization. Participants who died during the 2-year follow-up period were censored at the point of death, and participants who did not experience a hospitalization were censored at 2 years. There was no planned adjustment of *P* values for these outcomes, and they were viewed as either supportive or nonsupportive of the primary hypothesis. The level of significance was set at 1-sided *P* < .05. All statistical analyses were conducted using SAS software, version 9.4 (SAS Institute Inc).

## Results

**Figure 1.**
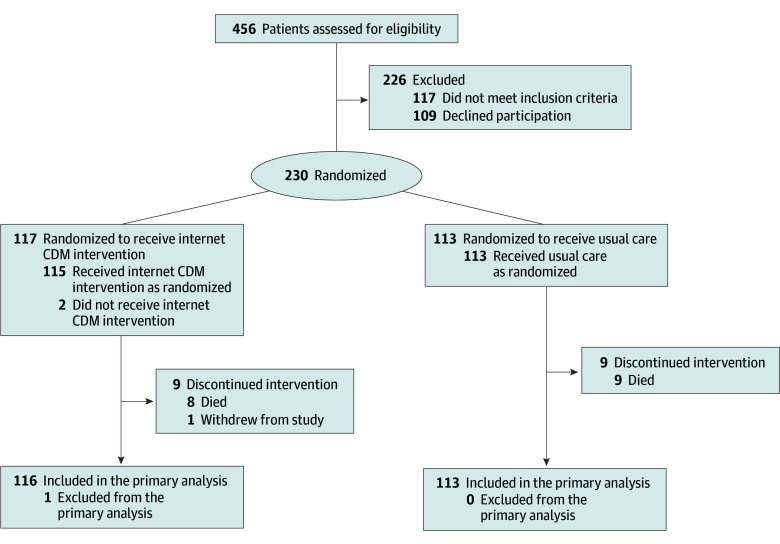
CONSORT Diagram

Between October 2011 and March 2015, 1431 PCPs were invited to participate via mail. Of those, 77 PCPs did not receive letters because they were sent to incorrect addresses, 789 did not respond, 185 did not have a primary care practice, 72 retired or were not currently practicing, and 6 declined to participate for unknown reasons. Of the remaining 311 eligible PCPs, 138 agreed to participate, and 125 mailed a total of 3438 invitation letters to patients. Among those invited, 456 patients (13.3%) contacted the research office and were screened for study eligibility. After exclusions, 230 patients referred by 71 PCPs were randomized (113 to the usual care group and 117 to the internet CDM group) (Figure 1). A total of 37 PCPs (52.1%) had patients enrolled in both the usual care and internet CDM groups. One participant in the internet CDM group withdrew from the study after randomization, resulting in 229 participants for whom data on the primary outcome were available.

Among 229 total participants, the mean (SD) age was 70.5 (9.1) years; 141 participants (61.6%) were male, and 88 (38.4%) were female (Table). Data on race and ethnicity were not collected because there was no planned analysis of these variables. The median number of target chronic diseases was 2 (25th and 75th percentiles, 2 and 3). The internet CDM and usual care groups were balanced with respect to age (mean [SD], 69.6 [8.8] years vs 71.3 [9.5] years, respectively), sex (eg, 72 of 116 men [62.1%] vs 69 of 113 men [61.1%]), number of chronic conditions (median, 2 conditions [25th and 75th percentiles, 2 and 3 conditions] for each group), daily internet access and use (eg, home or work: 115 of 116 participants [99.1%] vs 113 of 113 participants [100%]), educational level (eg, high school or equivalent: 33 of 114 participants [28.9%] vs 35 of 111 participants [31.5%]), and employment status (eg, retired: 82 of 114 participants [71.9%] vs 91 of 111 participants [82.0%]).

**Table.** Baseline Participant Characteristics

Characteristic	No./total No. (%)		
	All participants (N = 229)	Usual care group (n = 113)	Internet CDM group (n = 116)
Age, mean (SD), y	70.5 (9.1)	71.3 (9.5)	69.6 (8.8)
Sex			
Female	88/229 (38.4)	44/113 (38.9)	44/116 (37.9)
Male	141/229 (61.6)	69/113 (61.1)	72/116 (62.1)
Chronic disease			
Ischemic heart disease	135/229 (59.0)	66/113 (58.4)	69/116 (59.5)
Chronic heart failure	50/229 (21.8)	25/113 (22.1)	25/116 (21.6)
Diabetes	164/229 (71.6)	80/113 (70.8)	84/116 (72.4)
Chronic kidney disease	133/229 (58.1)	71/113 (62.8)	62/116 (53.4)
Chronic obstructive pulmonary disease	70/229 (30.6)	33/113 (29.2)	37/116 (31.9)
No. of chronic diseases, median (25th and 75th percentiles)	2 (2 and 3)	2 (2 and 3)	2 (2 and 3)
Daily internet access			
Home or work	228/229 (99.6)	113/113 (100)	115/116 (99.1)
Telephone	222/229 (96.9)	111/113 (98.2)	111/116 (95.7)
Smoking status			
Current	19/225 (8.4)	11/111 (9.9)	8/114 (7.0)
Former	146/225 (64.9)	74/111 (66.7)	72/114 (63.2)
Never	60/225 (26.7)	26/111 (23.4)	34/114 (29.8)
Total pretax household income, $			
<20 000	30/225 (13.3)	16/111 (14.4)	14/114 (12.3)
20 000-29 000	42/225 (18.7)	24/111 (21.6)	18/114 (15.8)
30 000-39 000	36/225 (16.0)	24/111 (21.6)	12/114 (10.5)
40 000-49 000	26/225 (11.6)	16/111 (14.4)	10/114 (8.8)
50 000-59 000	26/225 (12.4)	8/111 (7.2)	18/114 (15.8)
>59 000	49/225 (21.8)	14/111 (12.6)	35/114 (30.7)
Educational level			
<High school	42/225 (18.7)	22/111 (19.8)	20/114 (17.5)
High school or equivalent	68/225 (30.2)	35/111 (31.5)	33/114 (28.9)
Some postsecondary school	53/225 (23.6)	28/111 (25.2)	25/114 (21.9)
Postsecondary degree	50/225 (22.2)	22/111 (19.8)	28/114 (24.6)
Postgraduate degree	11/225 (4.9)	5/111 (4.5)	6/114 (5.3)
Current employment status			
Full-time	22/225 (9.8)	11/111 (9.9)	11/114 (9.6)
Part-time	6/225 (2.7)	2/111 (1.8)	4/114 (3.5)
Unemployed	6/225 (2.7)	3/111 (2.7)	3/114 (2.6)
Retired	173/225 (76.9)	91/111 (82.0)	82/114 (71.9)
Marital status			
Single	12/225 (5.3)	8/111 (7.2)	4/114 (3.5)
Married	153/225 (68.0)	73/111 (65.8)	80/114 (70.2)
Divorced	17/225 (7.6)	6/111 (5.4)	11/114 (9.6)
Widowed	34/225 (15.1)	19/111 (17.1)	15/114 (13.2)
Common law	8/225 (3.6)	3/111 (2.7)	5/114 (4.4)

Abbreviation: CDM, chronic disease management.

The median number of logins per week for the internet CDM group was 2.5 (25th and 75th percentiles, 1.2 and 3.1). A total of 98 of 116 participants (84.5%) logged in at least once per week. One participant did not engage with the intervention after randomization, and 1 participant discontinued intervention after 1 year. There were 2 protocol deviations (1.7%), in which participants did not wish to use the website; these participants were instead contacted by the nurse via telephone every 2 months. The remaining 112 participants (96.6%) in the internet CDM group completed the intervention protocol; however, all 116 participants in the group were included in the analysis. A total of 32 095 alerts were generated by the internet CDM group over the 2-year study period (median per participant, 201 alerts; range, 28-1247 alerts). Of those, 39 alerts from 25 unique participants resulted in referrals to participant PCPs, and 14 alerts from 2 unique participants resulted in referrals to the emergency department.

### Primary Outcome

**Figure 2.**
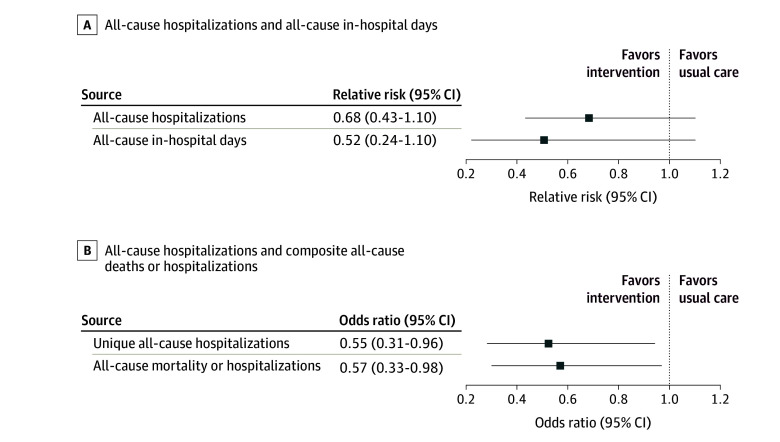
All-Cause Hospitalizations, All-Cause In-Hospital Days, and Composite of All-Cause Hospitalization or Death Models were adjusted for age, sex, and number of chronic conditions.

A total of 25 fewer all-cause hospitalizations occurred in the internet CDM group vs the usual care group (56 hospitalizations vs 81 hospitalizations; 30.9% reduction). Hospitalizations did not differ statistically between the 2 groups (relative risk [RR], 0.68; 95% CI, 0.43-1.10; *P* = .12) after adjusting for age, sex, and number of chronic conditions (Figure 2A). Excluding the 14 participants who died (8 in the internet CDM group and 6 in the usual care group) did not change the results (RR, 0.75; 95% CI, 0.47-1.21; *P* = .24).

### Secondary Outcomes

A total of 229 fewer in-hospital days occurred in the internet CDM group vs the usual care group (282 days vs 511 days). The number of in-hospital days did not differ significantly between groups (RR, 0.52; 95% CI, 0.24-1.10; *P* = .09) (Figure 2A).

There were no differences between the 2 groups with respect to changes in quality of life (as measured by the Medical Outcomes Study 36-item Short Form survey) (eTable 3 in Supplement 2). Self-management (as measured by the Health Education Impact Questionnaire) significantly changed in favor of the internet CDM intervention in 4 of the 8 domains: skill and technique acquisition, self-monitoring and insight, social integration and support, and emotional well-being (eTable 4 in Supplement 2). Social support (as measured by the Medical Outcomes Study Social Support Scale) significantly changed in favor of the internet CDM intervention in 2 of the 5 domains: emotional and informational support and overall support index (eTable 5 in Supplement 2).

### Exploratory Outcomes

**Figure 3.**
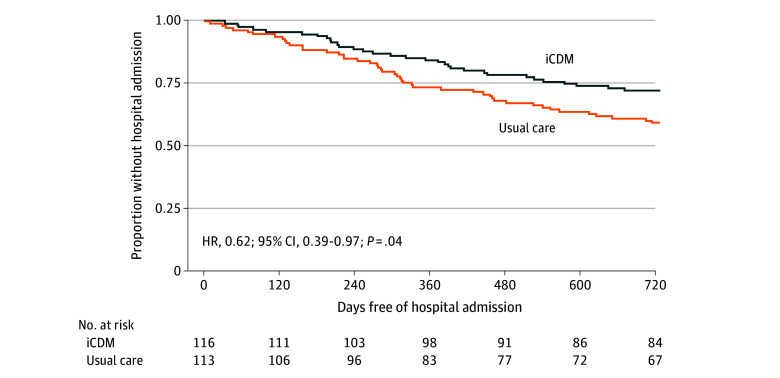
Time to First Hospitalization Models were adjusted for age, sex, and number of chronic conditions. HR indicates hazard ratio; and iCDM, internet chronic disease management.

Significantly fewer participants in the internet CDM group vs the usual care group had at least 1 all-cause hospitalization (32 of 116 individuals [27.6%] vs 46 of 113 individuals [40.7%]; odds ratio [OR], 0.55; 95% CI, 0.31-0.96; *P* = .03). In addition, the proportion of patients who experienced the composite outcome of all-cause hospitalization or death was lower in the internet CDM group vs the usual care group (37 of 116 individuals [31.9%] vs 51 of 113 individuals [45.1%]; OR, 0.57; 95% CI, 0.33-0.98; *P* = .04) (Figure 2B). Participants in the internet CDM groups had a lower risk of time to first hospitalization than those in the usual care group (hazard ratio, 0.62; 95% CI, 0.39-0.97; *P* = .04) (Figure 3).

## Discussion

This randomized clinical trial assessed the effect of an internet-based program on hospitalizations among patients with multimorbidity living in small urban and rural areas of British Columbia, Canada. The intervention resulted in a nonsignificant 30.9% reduction in all-cause hospitalizations along with improvements in self-management and social support. The proportion of participants admitted to hospital and the proportion who experienced the composite outcome of all-cause hospitalization or death were significantly lower among participants who received the internet CDM intervention vs usual care. The time to first hospitalization was also longer for participants in the intervention group.

The use of technology to support patients and PCPs in the management of chronic disease has increased and gained more prominence during the COVID-19 pandemic as health care organizations have sought to keep patients out of hospitals. Previous research has focused on examining either patients with a single disease or combinations of patients with single diseases. The Whole System Demonstrate study,^30^ which was a cluster-randomized investigation of a telehealth intervention among participants with diabetes, heart failure, or COPD, reported a small (13%) reduction in hospitalizations. A meta-analysis of randomized clinical trials of patients with heart failure found that telehealth interventions were associated with a reduction in hospitalizations of approximately 20% compared with usual care.^28^ Our internet-based intervention resulted in a 30.9% reduction in hospitalizations (56 hospitalizations in the internet CDM group vs 81 hospitalizations in the usual care group). These findings are consistent with data from a smaller study of 58 patients, in which 50% of participants had both heart failure and COPD; that study reported a nonsignificant 34% reduction in hospitalizations after 1 year.^27^ The study also reported a significantly lower proportion of patients in the intervention group were hospitalized,^27^ which is similar to our findings.

Consistent with interventions used in previous studies of patients with heart failure and COPD,^28,29^ the internet CDM program used an alert system to identify symptomatic participants who may have needed targeted care. In most cases, alerts were handled between the nurse and participant. As a result, participant self-management and social support significantly improved compared with usual care. In contrast to previous telemonitoring studies,^28,29^ we made use of participants’ own devices. This approach resulted in lower costs, fewer logistical issues with distribution, and less training while providing a model that could be readily scaled. The internet CDM intervention encouraged collaborative care among the participant, PCP, and nurse, ensuring a patient-centered decision-making process.^15,40^ These factors may explain the high level of engagement in the internet CDM intervention, with 96.6% of participants completing the full 2-year intervention and 84.5% accessing the website weekly. In addition, our program aligned with PCP fee structures to reduce barriers to active physician participation.^50^

We targeted patients in small urban and rural areas because they are generally the most underserved. However, this model of care can also be used to serve patients in urban areas who may not find it convenient to attend outpatient clinics or are limited to clinic waiting lists. In addition, remote models of care can minimize the need for patients with complex diseases to attend hospital-based outpatient clinics, reducing exposure to hospital-acquired infection and increasing the availability of hospital space and resources for those needing acute care. With the ongoing pandemic and resultant disruption in the care of patients with chronic conditions, it is expected that there will be increases in hospitalizations associated with chronic diseases.^24^ Digital health programs, such as the internet CDM intervention, can play a role in mitigating these increases.

### Strengths and Limitations

This study has several strengths. These strengths include its randomized design, low loss to follow-up, and high uptake of the intervention. In addition, the intervention was integrated with and informed by primary care, reflecting real-world implementation. The exclusive use of remote communication made the program ideal for patient monitoring across geographical regions.

The study also has limitations. The main limitation was its inability to reach the target sample of 300 participants. One barrier to PCP participation was the requirement to screen patient records for study inclusion criteria. At the time of study initiation, fewer than 30% of PCPs used electronic medical records, and PCPs without access to electronic medical records did not wish to manually screen patient records and therefore did not choose to participate. With the use of electronic medical records now higher than 86%,^51^ we expect that this increase may lead to greater PCP uptake. Participant recruitment may also have been limited by ethical requirements to restrict initial contact with patients to mail only. It is well known that face-to-face recruitment for study participation is more effective and has been reported to be 3 times more effective than recruitment by mail.^52^ In addition, the present study was not a cluster-randomized clinical trial, and 52.1% of participating PCPs had patients enrolled in both study arms. However, because participants in the usual care group did not have access to the internet CDM intervention, the potential for contamination of data was minimal.

## Conclusions

In this randomized clinical trial, a CDM program comprising internet-based self-management and symptom monitoring integrated within primary care resulted in a nonsignificant reduction in all-cause hospitalizations among patients with multimorbidity. In addition, among participants who received the internet CDM intervention, fewer were admitted to the hospital, and fewer experienced the composite outcome of all-cause hospitalization or death compared with those who received usual care. The time to first hospitalization was also longer in the intervention group compared with the usual care group. Future research to address the cost-effectiveness of multimorbidity programs is warranted. An internet-based self-management and symptom monitoring program integrated with primary care has the potential to augment care for patients with multimorbidity.

## Notes

Supplement 1.
Trial Protocol

Supplement 2. **eTable 1.** Intervention Participant Symptom-Related Questions and Biometric Data to Enter for Each of the 5 Chronic Diseases **eTable 2.** Default Alert Thresholds for Biometric Data **eTable 3.** Baseline and Follow-up Values of Quality of Life Using the Medical Outcomes Study 36-Item Short Form Survey, Version 2 **eTable 4.** Baseline and Follow-up Values of Self-Management Measures Using the Health Education Impact Questionnaire **eTable 5.** Baseline and Follow-up Values of Social Support Using the Medical Outcomes Study Social Support Scale
eReferences

Supplement 3.
Data Sharing Statement
