# Supplementary material for: Assessment of an Interactive Digital Health–Based Self-management Program to Reduce Hospitalizations Among Patients With Multiple Chronic Diseases: A Randomized Clinical Trial
Source: JAMA Netw Open. 2021 Dec 28;4(12):e2140591. doi: 10.1001/jamanetworkopen.2021.40591 (PMC12243620; doi:10.1001/jamanetworkopen.2021.40591)
Supplement: Supplement 1. — Trial Protocol [file jamanetwopen-e2140591-s001.pdf]

RESEARCH PROPOSAL
Utilization of an interactive Internet-based platform for managing chronic diseases at a distance (iCDM)
Dr. Scott Lear

TABLE OF CONTENTS

Abstract	2
Background Information	2
Burden and Management of Chronic Diseases	2
The Patient with Multiple Chronic Diseases	3
Telehealth in Chronic Disease Management	3
Justification for Study	3
Research Program	5
Purpose of Study	5
Hypothesis	5
Study Population	5
Subject Inclusion Criteria	5
Subject Exclusion Criteria	5
Study Outcomes	5
Sample Size	6
Baseline Assessment	6
Randomization	6
Security and Privacy Issues	7
Follow-up Assessments	7
Methodology of Study Outcomes and Measures	8
Healthcare Utilization	8
Psychometric Measures	8
Patient and Provider Experience and Satisfaction	8
Adherence to the iCDM	8
Additional Variables Assessed at Baseline	8
Statistical Analyses	8
Study Considerations	9
Self-reported Measures	9
Cross-group Treatment Contamination	9
Subject Safety Provisions	9
Participant Confidentiality and Handling of Data	9
Ensuring Safety and Minimizing Risk	9
Participant Safety Monitoring, Reporting and Stopping Rules	9
Dissemination of Outcomes	10
Impact and Future Directions	10
Appendices – iCDM Overview	11
References	23

ABSTRACT
Chronic diseases, those diseases that are managed and cannot be cured, are becoming an increasing burden to the Canadian healthcare system. Chronic diseases that have especially high impact include ischemic heart disease, heart failure, diabetes, chronic kidney disease and chronic lung diseases. Patients with these types of chronic conditions account for a substantial proportion of hospitalizations and premature mortality. Many patients have more than one of these chronic diseases (e.g., diabetes and kidney disease), which pose unique challenges because these individuals are more likely to develop complications. For patients who live in remote, rural and small urban areas that do not have access to specialized outpatient chronic disease management programs such as cardiac rehabilitation, diabetes teaching centres, COPD clinics, etc. (henceforth referred to as 'rural'), they are especially vulnerable to the consequences of chronic diseases due to reduced access to healthcare. These patients create additional complexities for healthcare as they present with greater risk factors, hospitalization rates and mortality rates from chronic diseases than urban patients.

Traditionally, healthcare management of patients who have multiple chronic health conditions is based on disease-specific strategies that are usually independent of one another. This often leads to fragmented and episodic care, resulting in increased patient burden to navigate multiple providers and systems, along with duplication of tests and healthcare inefficiencies. However, patients in rural areas often do not have the opportunity to receive even this level of care. Effective management of these patients requires multi-disciplinary coordinated care between patients, primary care physicians (PCP) and specialist providers (physicians and allied health professionals). Tools to facilitate this coordination have not been formally developed nor tested in a robust manner.

We propose to develop and evaluate a multi-chronic disease management program delivered through the Internet (with telephone supports) focused on high-impact chronic diseases targeted to patients in rural communities and at the highest risk of further marginalization.

This study will consist of a single-blinded randomized controlled trial to investigate the efficacy of the iCDM in 318 patients with two or more of the target chronic diseases living in rural areas. This study will adhere to the CONSORT guidelines for RCT reporting and be registered at www.clinicaltrials.gov. Within this Aim, we will be able to address the following research questions:

Q1. What is the effect of iCDM on healthcare utilization and patient self-management outcomes?
Q2. What is the long-term compliance to the iCDM?
Q3. What is the level of patient and provider satisfaction?

BACKGROUND INFORMATION

Burden and Management of Chronic Diseases: In 2005, more one-third of Canadians were burdened with one or more chronic diseases.[1] Moreover, it is estimated that, between 2005 and 2015, two million Canadians will die of causes related to a chronic disease at a cost of more than $9 billion of national income.[2] These trends are likely to continue: over the next 20-30 years, as the proportion of people aged >65 years will be greater than that of other age groups in Canada, and the prevalence of chronic diseases is expected to rise in parallel. Those with a chronic disease use twice the amount of healthcare resources compared to the average adult and have hospital stays that are four times longer than those with acute conditions.[3] According to the Health Indicators 2008 Report, hospitalizations for chronic diseases are ~60% higher per capita in rural areas than in urban areas. In this context, the CIHI report "*How Healthy are Rural Canadians*" (2006) indicated that rural Canadians have a greater prevalence of risk factors that contribute to or exacerbate chronic conditions (e.g., poor nutrition, smoking and obesity) and higher mortality rates from chronic diseases.[4] The report concluded that

> "programs aimed at secondary prevention of chronic diseases … are key to population health. The concentration of health resources, expertise, technologies and services in larger urban centres, together with the challenges of rural transportation, may have made such services less accessible to those living in smaller or more remote communities."

The management of patients with chronic diseases requires that primary care services are complemented by information supports, patient self-management and a multi-disciplinary team based approach including specialized services. This approach is outlined in BC's Expanded Chronic Care Model. Application of this model entails a shift of focus from primarily episodic interventions to include proactive care and improved support for the management of the day-to-day problems faced by people living with chronic disease. Programs that follow this model show improved patient outcomes and reduced healthcare utilization; they are cost-effective and are recommended in national guidelines for disease management. These programs also work to improve attachment

between the patient and their PCP, which has also been demonstrated to reduce healthcare costs in these patients.[5]

To improve access to care for rural populations, healthcare decision makers need new models of service delivery that are based on the principles of chronic disease management, and respond to the needs of rural patients and complement primary care.

The Patient with Multiple Chronic Diseases: Patients with one chronic disease often have, or are at risk for, another chronic disease. For example, diabetes and chronic kidney disease are risk factors for ischemic heart disease, and many patients with ischemic heart disease will face a future diagnosis of heart failure.[6] This group of complex patients represents a substantial challenge to healthcare resources; we have found that compared to patients with only one chronic disease, these patients present with a greater number of primary care and specialist visits, acute care hospital days, and redundant duplicate laboratory tests. The following scenarios highlight the challenges faced by patients with multiple diseases living in urban and rural areas:

- Urban scenario: Mrs S lives in Vancouver and was diagnosed with diabetes 10 years ago. She attends a diabetes teaching centre in which she consults with an endocrinologist, nurse and dietitian. She also has impaired renal function and attends her local renal clinic where she sees a nephrologist, a second nurse and a second dietitian. Recently, Mrs S had a myocardial infarction and was referred to the hospital cardiac rehabilitation program, where she will consult with a cardiologist, a third nurse, a third dietitian and an exercise specialist. While each clinic is aware of Mrs S's co-morbidities, her care is not coordinated: each discipline (endocrine vs renal vs cardiac rehab) is focused on its own disease outcomes and prognosis. The clinics are not located in physical proximity and there is no opportunity for sharing medical records. This scenario results in an excessive burden of time and resource allocation, in parallel with potential for confusion among the patient and healthcare providers, and duplication of medical tests. The overall effect is healthcare inefficiencies and risk of serious mistakes, owing to a lack of a shared patient care plan.
- Rural scenario: Mr. J has chronic obstructive pulmonary disease (COPD) and requires cardiac surgery. He lives in rural BC; for his surgery, he is transferred to Vancouver and afterwards discharged to home. Post-operatively he sees his family physician, but there are no cardiologists in his area. In addition, he cannot access the specialized services in the Vancouver area that are essential for long-term management. His local primary care provider can assist Mr. J but cannot overcome many of the issues he is likely to face. As Mr J cannot access appropriate care, he ignores his condition until it progresses to the point where more complex and costly care is needed, and he is re-admitted to hospital in Vancouver.

Telehealth in Chronic Disease Management: For patients in rural communities, the opportunity to attend ambulatory care clinics is not always an option, or only at substantial cost to the patient with respect to time and travel to urban centres. Additionally, the opportunity for rural patients to receive quality care close to, or within their homes, is of great benefit as it reduces the need for extensive travel (sometimes on hazardous rural roads) and the potential burden of clinic visits. The use of telehealth[1] has been identified as an effective modality for chronic disease management[7-12] and is actively promoted by national organizations as having great promise for health service delivery in rural areas.[13-15] Numerous systematic reviews have highlighted the potential benefits of using telehealth to assist with the management of patients with chronic diseases. For example, a meta-analysis of 14 randomized studies of remote monitoring of heart failure patients reported a 21% reduction in HF hospital admissions and a 20% reduction in all-cause mortality.[11] Other reviews from studies across a range of chronic diseases, such as diabetes and pulmonary diseases have reported benefits regarding patient acceptance, improved lifestyle behaviours, clinical outcomes and reduced healthcare utilization.[8-10, 12, 16-19] The most effective telehealth programs were those that incorporated patient monitoring and nurse telephone support.[17]

JUSTIFICATION FOR STUDY

The Internet as an Innovative Medium for Chronic Disease Management: The Internet as a mode for healthcare delivery has numerous advantages: 1) it is ubiquitous with increasing access in all age groups, 2) it is inexpensive, 3) it facilitates both patient data transfer and patient feedback, thereby supporting patient self-management, 4) it is scalable to large patient volumes, 5) it delivers health care directly to the patient (predominantly in their home) and 6) it requires minimal set-up for patients with current Internet access.

In addition to our own work, other studies have also indicated that the Internet can be used to support patient self-management and monitoring in patients with heart failure (reviewed by our group[20]),[21,22-26] ischemic heart disease,[8, 27] diabetes,[28-33] chronic kidney disease[34] and chronic obstructive pulmonary disease.[35-36] Many of

[1] Telehealth refers to use of technology such as the telephone, Internet, video conferencing and telemonitoring.

these studies have focused on patient acceptance and feasibility demonstrating that patients with chronic disease are willing to enter self-measured data online and communicate with health care providers via email and/or chat/video conferencing. A few studies have indicated that Internet interventions can be effective in improving self-management skills, managing risk factors and improving symptoms but these are still limited to sample sizes below 100 patients with limited outcome measures.[8, 27, 37-44]

Many reviews however, noted that findings are not always consistent due to studies being of poor quality, with no control group and/or small sample sizes. This is highlighted in a review of 79 studies analyzed for methodological aspects that found most had sample sizes below 100 and only 11% of studies were randomized.[45] This is a concern as non-randomized studies tend to have more positive outcomes.[46] Additionally, many studies were short-term, with very few lasting beyond a year; as a result, studies are needed to assess the long-term benefits of telehealth interventions.[16]

While the use of the Internet holds promise for patient-focused chronic disease management[7, 47]; environmental scans of telehealth service initiatives in Canada focus primarily on consultation services that require sophisticated and costly video conferencing networks situated in hospitals or clinics.[48-49] This is in spite of the federal funding Canada Infoway receives.[50] This may be due to a lack of evidence from good quality studies on clinical care and self-management as emphasized in reviews and opinion pieces.[47, 51-54] In fact, Canada lags far behind the United States, United Kingdom and Japan with respect to telehealth research.[54] This lack of robust evidence makes it difficult for decision-makers to make informed decisions and has been cited by Wotton et al. for the poor uptake of telehealth solutions.[55]

Given the lack of robust evidence, Canada's diverse geography and universal health care system, research studies specifically designed to demonstrate any potential treatment effects and contextualized in the Canadian environment are needed to inform health care. A randomized study is therefore needed to address the deficiencies in previous studies to effectively evaluate the efficacy of an Internet-based self-management program compared to usual care and inform decision-makers in healthcare.

The proposed study will address the deficiencies of previous studies by:

- Testing the efficacy of an Internet-based intervention compared to usual care using randomization
- Evaluating a sample size of over 300 participants
- Conducting the study over a two-year time frame
- Using robust outcome measures of healthcare utilization and quality of life
- Involving healthcare decision-makers in the design of the study methodology and intervention
- Evaluating the intervention in the context of Canadian healthcare

As the proposed study's intervention is focused on supporting patient self-management and alerting the patient's primary care provider to deterioration in their patient's health, we foresee the intervention to be of low risk. This is supported by the finding that none of the previously mentioned studies have reported adverse effects or patient risks as a consequence of using Internet-based self-management programs.

RESEARCH PROGRAM

Purpose of Study: We propose to develop and evaluate a multi-chronic disease management program delivered through the Internet (with telephone supports) focused on high-impact chronic diseases targeted to patients in rural communities and at the highest risk of further marginalization.

Hypothesis

Patients with multiple chronic diseases utilizing a chronic disease management program delivered through the Internet (with telephone supports) will have lower hospital admissions over a two-year period compared to patients utilizing usual care only.

This study will consist of a single-blinded randomized controlled trial to investigate the efficacy of the iCDM in 318 patients with two or more of the target chronic diseases living in rural areas. This study will adhere to the CONSORT guidelines for RCT reporting and has been registered at www.clinicaltrials.gov (registration # NCT01342263). Within this Aim, we will be able to address the following research questions:

Q1. What is the effect of iCDM on healthcare utilization and patient self-management outcomes?
Q2. What is the long-term compliance to the iCDM?
Q3. What is the level of patient and provider satisfaction?

Study Population: We will recruit patients through primary care networks and other practicing primary care physicians and nurse practitioners. Physicians and nurse practitioners will be approached in Fraser Health, ~~(through Chilliwack Division of Family Practice),~~ Northern Health, Interior Health, ~~and~~ Vancouver Coastal Health and Vancouver Island Health who practice outside of major urban areas. These areas have been identified based on the lack of access for patients to specialized chronic disease outpatient programs. We will approach these physicians and nurse practitioners by first sending a letter to the physician/nurse practitioner briefly outlining the study and asking for their support in recruiting patients from their practice. This letter will be jointly sent from Dr. Scott Lear and one of the physician leaders in the area (see attached letter). Many of physicians and nurse practitioners we will be approaching have electronic medical records (EMR). In those cases, we will ask the physician/nurse practitioner or another individual who has approved access to the EMR to identify potentially eligible patients (based on the disease and age criteria listed below). Physicians and nurse practitioners who do not respond to the initial letter within 10 days of the letter being sent will be sent a follow-up email from the physician leader with an electronic version of the letter and indicating that the local research coordinator will contact them by telephone to follow-up with any questions/clarifications about the study. For physicians and nurse practitioners in Interior Health, Northern Health ~~and~~ Vancouver Coastal Health and Vancouver Island Health who agree to support recruitment of their patients, we will draft a letter for the physician/nurse practitioner to send to those potentially eligible patients inviting them to participate in the study by contacting the study coordinator (see attached letter IH/NH/VCH,VIH). The letter will be sent by the physician/nurse practitioner and include a copy of the patient consent form. Once the patient has contacted the research coordinator, he/she will be screened further for study eligibility and have the opportunity to ask questions regarding the study. The research coordinator will explain the consent form and obtain informed consent (this method has already received approval from the Northern Health Authority, Interior Health Authority and Vancouver Coastal Health Authority REBs). For physicians and nurse practitioners in Fraser Health who agree to support recruitment of their patients, we will draft a letter for the physician/nurse practitioner to send to those potentially eligible patients informing them briefly of the study and asking the patient for consent for the family physician/nurse practitioner to release their contact information to the research coordinator (see attached letter for release of contact information). For those patients who consent to have their contact information released to the research coordinator, the research coordinator will mail a letter of invite for the study along with the consent form (see attached letter of invite). After 10 days of the letter being mailed, the research coordinator will telephone the patient to address any questions they may have regarding the study. In addition, we will also place study brochures and posters in the physicians'/nurse practitioners' offices that can be handed out by the physician/nurse practitioner or their office assistant directing interested patients to contact the study coordinator (see attached brochure/poster). Physicians/nurse practitioners and their offices will be compensated for their time in supporting recruitment of their patients (ie: screening of their patients, sending out letters, etc.). There will be no costs to the patients to participate in this study.

Inclusion Criteria: Men and women with two or more of the five targeted chronic diseases; daily Internet access (home, work or other environment) by any means (such as personal computer, personal data assistant or smartphone); over 19 years of age; and able to read, write and understand English without difficulty.
Exclusion Criteria: Patients with significant co-morbidities (excluding the five targeted conditions these include HIV, uncontrolled metabolic/endocrine disorders, prior stroke or other conditions affecting cognitive abilities to

provide consent and participate, alcohol/drug addiction) that may interfere with effective management; patients who have scheduled surgical procedures; and patients who are unable to provide informed consent**.**

Detailed records will be kept of all individuals who are screened for the study to clearly define the population from which our study cohort will be derived and to ensure our reporting will follow the CONSORT guidelines.

Study Outcomes: *The primary outcome will be the difference between the iCDM and usual care groups in hospital admissions from the time of randomization to the end of two years.* Data will be collected by participant self-report and confirmed though access to participant medical records in those who sign the study's Medical Records Release Form. We will also collect data from Provincial Databases hosted by the Population Data BC; Medical Services Plan payment information file, PharmaCare file, hospital separations file and Vital Statistics deaths and clinical deaths on secondary outcomes of healthcare utilization such as length of hospital admissions, emergency room visits, physician/nurse practitioner visits, diagnostic and lab procedures. In addition, secondary outcomes will include patient quality of life, self-management, patient and provider experience, and mortality (see below).

Sample Size Determination: We have identified a convenience sample of 318 participants as feasible based on our methods of recruitment and study timelines. Based on our current study of a 'virtual cardiac rehabilitation program' in Northern BC which has a reported drop-out rate of 12%, we have set a drop-out rate of 15% due to patient withdrawal, death and moving outside of the province, and will therefore finish with 270 participants. We will collect detailed information on the subjects who withdraw and their reasons for withdrawing. In our pilot combined clinic at St. Paul's Hospital for patients with two or more of cardiovascular disease, diabetes and chronic kidney disease, we observed 32 hospitalizations over a two-year period in the 30 usual care patients (a baseline event rate of 107%) compared to 24 hospitalizations over a two-year period in 34 patients in the combined pilot clinic (event rate of 71%) resulting in a 33.6% reduction in events. We have based the current study power estimates on a 25% reduction in hospital admissions by the iCDM. We have chosen a more conservative effect of the iCDM as the iCDM is directed more at supporting patient self-management and monitoring, and does not involve direct medical management. Based on a 25% reduction in hospital admissions, and an alpha of 0.05, we have outlined the power given the following estimated event rates using the method of Demidenko E[56-57] in the table below. We have provided a range of event rates as we do not have documented event rates from our targeted patient population. If the event rate is similar to that observed in our pilot clinic at St. Paul's Hospital, we will have sufficient baseline events for a power greater than 0.80 to detect an effect of 25% reduction in events.

*Usual Care**	*iCDM**	*Power*
40%	30%	0.39
50%	38%	0.55
60%	45%	0.67
70%	53%	0.78

Baseline Assessment: We will collect the following data variables on all consenting participants: social demographics, medical history, current medications, self-management and quality of life, smoking status, alcohol consumption, e-health literacy, and recent diagnostic and lab tests (<6 months). The baseline (and follow-up) assessment will be conducted without the need for the research coordinator to meet with the patient. Data on patient demographics, medical history, current medications, smoking status, and recent diagnostic and lab tests will be abstracted from the patient's physician's/nurse practitioner's medical record and confirmed by telephone with the patient. For all other outcomes we will be using self-administered questionnaires which will be mailed to the patient with a stamped return envelope to mail back. If after two weeks the questionnaires have not been received, the research coordinator will contact the patient by telephone to follow-up. We will create a toll-free number for participants to call the research coordinator to return any messages from the coordinator and ask questions regarding assessment data collection so that participants will not have to incur long-distance phone charges.

Randomization: Participants will be stratified by sex and site of recruitment (based on health authority), and then randomized to either usual care or the iCDM using computer-generated variable blocks of 2, 4 and 6 within a web-based randomization system. A research assistant not involved in data collection will access the randomization system using a unique authorization code. The date and time, patient identifiers and randomization codes will automatically be maintained in a transaction file. This same research assistant will be responsible for communicating group assignments to the study participants. Participants will be informed not to discuss their group assignment with the research coordinator responsible for data collection to ensure the study

remains single-blinded. If at the time of being informed of their group assignment, the participant becomes distressed, anxious or upset, the research coordinator will inform the participant that the study psychologist is available to discuss their concerns if they wish.

Usual Care Group: Participants randomized to usual care will be given educational information regarding general chronic disease management and a list of Internet-based resources. There are no formal specialized ambulatory care clinics in the areas in which our target participants reside. Therefore, the overwhelming majority of patients are managed exclusively by their family physician. In some cases, patients may be referred to a local internist and may also have the opportunity to contact local community nurses for guidance on their care. Participants in the usual care group will be free to seek treatment as they wish and as their clinical providers see appropriate. There will be no contact between the study personnel and the usual care participants for the duration of the study, or any attempt to control for the level of patient care. If at the end of the study the results indicate superiority of the intervention, the participants in the usual care group will be offered the vHFC intervention for a three-month period.

Intervention Group: The iCDM is a nurse-supervised patient self-management and monitoring system that has been designed by a clinical advisory committee that includes specialist, family physician, allied health and patient representation (see Appendices for greater detail). The iCDM is meant to support patients and their family physicians/nurse practitioners in their care and not to supplant it.

Upon randomization, a letter will be sent to the patient's primary care provider (PCP) from the study investigators informing them of their patient's participation in the study, a brief description of the study. The iCDM nurse will contact the participant's family physician/nurse practitioner[2] to follow-up on the letter, discuss the intervention protocol and preferred method of communication, as well as to offer them access to the iCDM to view their patient's progress. Throughout the intervention, the iCDM nurse will communicate with the participants' family physicians/nurse practitioners to work collaboratively on participant care/action plans. Care plan development and communication with family physicians/nurse practitioners will be respectful of the family physician's/nurse practitioner's time and cognizant of any existing supports put in place for such purposes.

It is anticipated that family physician/nurse practitioner engagement with the iCDM will differ from physician/nurse practitioner to physician/nurse practitioner. However, we believe the benefits to the participant and the family physician/nurse practitioner will be greater through active involvement of the family physician/nurse practitioner. The family physician/nurse practitioner will remain ultimately responsible for their patient's care and authorizing any care/action plans. The iCDM nurse will only be involved in medical management of the participant if authorized by the family physician/nurse practitioner through care/action plans.

Participants will be registered to the iCDM with a unique username and password by the research assistant who conducted the randomization process (see Appendix for description of iCDM). Participants will be given the link to the iCDM, a simple manual on how to use the iCDM (which will also be on the iCDM program) and trained on the system over the telephone by the research assistant. Participants will have access to the iCDM for a two year period. A toll free technical support line for the participants will also be available.

The iCDM nurse has been hired and has experience with telephone management of patients and behavioural change (previous telehealth smoking-cessation nurse) and chronic disease management. She has participated in the cardiac, diabetes, nephrology and COPD outpatient clinics for a two-month period to enhance her skills in those disease areas. The iCDM nurse will review patient data, communicate with the participants, implement appropriate care and/or referral, and interact with the participants' PCP. Participants will also be able to interact with a dietitian and exercise specialist to support them in their disease management. Participants in the intervention group will be free to seek treatment as they wish and as their clinical providers see appropriate.

Security and Privacy Issues: The iCDM will be developed with the utmost attention to compliance with BC Privacy Legislation, security requirements of our partner health authorities. The iCDM program will be password protected and hosted on a standalone secure server. The website will be housed on a dedicated server. All data transmissions to and from the web server will be conducted via an SSL Internet connection to ensure encryption of transmitted data (similar to online banking). During the consent procedure, potential participants will be informed of their security and privacy rights as well as our efforts to ensure that these are maintained.

Follow-up Assessments (both groups): One year post-randomization, participants will be contacted by the research coordinator by telephone to administer the EuroQol EQ-5D-5L questionnaire and to ask simple

[2] Given our method of recruitment through physician offices and ambulatory care clinics, we anticipate our patient pool will be derived from practices where physicians will be informed and supportive of the study.

questions regarding their health in the past year to assess any adverse events. This will consist of an initial question asking the participant if they have been admitted to hospital for any reason in the previous year. If the participant answers 'yes' then additional questions on the nature and location of the event will be collected in order to report the event to the overseeing REBs (see below). If no contact has been made after a total of three unresponsive telephone calls the participant's family physician/nurse practitioner will be contacted to assess the vital status of the participant. If the physician/nurse practitioner indicates the participant is alive, we will confirm their contact details and proceed with sending a letter to the participant. If a response is not received within 10 days, the participant's alternate contact will be telephoned (this will have been collected at the time of participant consent). If the physician/nurse practitioner indicates the participant has died, we will collect the appropriate medical records for reporting the adverse event to the overseeing REBs.

After two years, the research coordinator will contact participants to inform them of their end of study follow-up assessment and assess adverse events similar to the one-year telephone contact above. This will consist of an assessment for secondary outcomes similar to the baseline assessment. Data on patient demographics, medical history, current medications, smoking status, and recent diagnostic and lab tests will be abstracted from the patient's physician's/nurse practitioner's medical record and confirmed by telephone with the patient. Questionnaires will be mailed to the patient with a stamped return envelope to mail back to the research centre with follow-up by the research coordinator as outlined above.

The research coordinators' roles will be limited to patient screening, participant recruitment, participant assessment and reporting of adverse events only. They will not be involved in delivering the iCDM intervention or in the care of the study participants.

METHODOLOGY OF STUDY OUTCOMES AND MEASURES

Healthcare Utilization: Data (hospital admissions, length of hospital stay, emergency room visits, physician/nurse practitioner visits, diagnostic and lab procedures) will be collected by participant self-report and confirmed though access to participant medical records in those who sign the study's Medical Records Release Form. We will also access provincial ministry databases at Population Data BC. *Mortality* will be assessed by accessing the Population Data BC database. We will also assess the amount of time the iCDM health professionals engage with the program and the participants so that we will be able to estimate patient loads that can be managed for future implementation.

Psychometric Measures: *Quality of Life* will be assessed by the Medical Outcomes Study 36-item Short Form survey.[58-59] This is a widely-used, self-administered instrument that provides indices of various components of the construct of "quality of life": patient health, patient ability to perform certain tasks and any adjustments they had to make. *Self-management* will be assessed by the Health Education Impact Questionnaire (heiQ) which is used for assessing patient education and self-management in participants with chronic conditions.[60] *Social support* will be assessed using the Medical Outcomes Study Social Support Scale in which participants report their level of perceived support.[61] The EuroQol EQ-5D-5L health questionnaire will be used to assess *health outcomes.*[62] We will use data collected from it to perform a cost-utility analysis that takes into account benefits experienced by study participants.

Patient and Provider Experience and Satisfaction: Participants enrolled in the iCDM intervention will undergo a semi-structured, open-ended interview at the end of the intervention to explore patient experiences and to increase our understanding of patient factors that influence acceptance and use. These interviews will be transcribed and entered into the qualitative research software NVivo 7 (QRS International, Victoria, Australia) which will be used to manage data and to provide a structure for analysis. A three-stage analysis of the transcript data (open-coding, axial coding, and selective coding) will be guided by an inductive iterative approach.[63-64] Participants for the interview will be identified by stratified purposeful sampling[65] with representation from both sexes and include participants from each recruitment method. The final number of interviews will be driven by the prospective discovery of factors (or concepts) that may affect participant satisfaction with care provided through iCDM. Interview transcripts will have personal identifiers removed and a double coding system will be used to link the data with the interviewee. In a similar fashion, we will interview family physicians/nurse practitioners of iCDM participants to explore PCP experience including satisfaction, attitudes and feedback. Participating physicians/nurse practitioners will be reimbursed for their time to ensure participation. Together, these interviews will help to elicit understanding of the role and meaning of the iCDM within the management these participants.

Adherence to the iCDM will be assessed through website use (logins) and completion of data entry. We will track which areas of the iCDM are frequented by the participants so that we can better understand which elements of the iCDM were most beneficial to participants. We will also assess the number and nature of alerts

generated per patient to provide insight into areas where participants need further support with managing their condition.

Additional Variables Assessed at Baseline: *e-Health Literacy* will be assessed using the eHealth Literacy Scale (eHEALS) in all participants during their baseline assessment only.[66] The eHEALS is used to assess the combined knowledge, comfort, and perceived skills at finding, evaluating, and applying electronic health information to health problems. *Depression* will be assessed using the Center for Epidemiologic Studies Depression scale.[67] These data will be used as potential independent determinants to identify what patient characteristics are associated with improved outcomes.

STATISTICAL ANALYSES

We anticipate that 10% of the study population will be lost-to-follow-up at the end of study. Lost-to-follow-up will be defined as those participants who have died, withdrawn from the study or moved out of the province. Every effort will be made to obtain final data on the primary and secondary outcomes from subjects regardless of the extent of study participation to that point. *Lack of compliance to the iCDM will not be a reason for withdrawal from the study.* We will explore differences in baseline variables between those lost-to-follow-up and those who remain in the study.

The primary outcome of hospital admissions between the two groups from baseline to two (dependent variable) years will be modeled using ordered logistic regression with group assignment, age and sex as *a priori* covariates. In the event of potentially clinically relevant differences in baseline factors, we will include these covariates in the model (such as socio-demographics, e-health literacy, depression, and other morbidities). Non-normally distributed continuous variables will be transformed prior to analyses. Residual plots for the primary outcome models will be plotted and assessed for model adequacy. Secondary outcomes between the two groups will be compared using the Pearson Chi-square test for categorical variables and independent samples t-test for continuous variables. All analyses will be governed by intent-to-treat with no group crossovers.

STUDY CONSIDERATIONS

Self-reported Measures: The iCDM will rely on patient self-reported data entry (i.e. participants entering their blood sugar reading from their glucometer). There is a potential for patient error when entering the data. We will safeguard against this possibility by requiring confirmation of data entry and by imposing limits for which data will not be accepted (i.e. a systolic blood pressure of 1300 will not be allowed to be entered). The use of self-reported measures is an important activity to engage the patient in the management of their care. Indeed, there are numerous models in which physicians/nurse practitioners rely on patient self-report such as reporting of symptoms, medication use and home blood sugar values to name a few.

Cross-group Treatment Contamination: As we will be recruiting through PCP offices and the unit of randomization will be the participant, it is possible that one physician/nurse practitioner may have more than one patient in the study and these patients are randomized to different groups. This could lead to possible treatment contamination in which the physician/nurse practitioner alters the treatment of the usual care patient based on their involvement with the treatment of the iCDM patient. However, we believe this possibility to have negligible, if any, effect on the study outcomes as patient management decisions by the PCP due to their patient's enrolment in the iCDM will be initiated as a result of the patient's engagement with the iCDM. This will not happen in the usual care group as they are not undergoing supported self-management or monitoring to alert the PCP of any untoward conditions. In addition, in many cases, alerts generated by an iCDM patient will not require the PCP's involvement.

SUBJECT SAFETY PROVISIONS

Participant confidentiality and handling of data: Any information that is obtained during this study will be kept confidential to the full extent permitted by the law. We will not need to record participant's name or personal identifiers (ie: date of birth, personal health number) on any study material, which will be labeled with an anonymous code number. The interviews will be audio-recorded and transcribed, and held over the phone. All interviewees will be instructed not to provide their name or any identifiers that could be used to identify themselves or others prior to beginning the audio recording. The audio recording will be destroyed after being transcribed. Data will be entered into a secure database. Only the main contact, the research investigators, the study research assistants, and the research coordinators will have access to this data. We will keep all the study materials in the locked filing cabinet in the locked office of the research coordinators in Prince George and Vancouver. Upon completion of the study, the hardcopies and electronic data will then be couriered to the main study office located at St. Paul's Hospital for 6 years after publication. We will destroy the data at the end of the storage period.

Ensuring safety and minimizing risk: The risks for participating in this study are minimal. As there is no interference/control of care for participants in the usual care group, we do not anticipate any additional risk associated with participation. While we anticipate participants in the iCDM intervention group will benefit from the additional monitoring, we recognize the potential for some participants to interpret the intervention as a replacement for their treatment, which is not the intention. To minimize this potential risk, we have clearly outlined on the consent form that the iCDM intervention does not replace medical treatment. Participants will be informed of this again when they are trained on the use of the iCDM and they will see a similar message upon first logging on to the website that they first must read and agree to before proceeding to the other areas of the website. Lastly, if a participant generates and 'alert' the alert message will also indicate that if they feel unwell they should seek medical attention.

Participant Safety Monitoring, Reporting and Stopping Rules: There will be two time periods at which participant adverse events will be assessed; this will occur one-year following randomization and at study completion at two years (see Follow-up Assessments above). Adverse events (hospitalizations and deaths) will be reported to the overseeing REBs as they are identified. Two interim analyses of efficacy focused on the primary outcome of hospitalization or death will be performed; the first after all participants have completed the one-year follow-up, and the second once half the patients have completed the two year follow-up. The analyses will be examined by an independent data and safety monitoring committee (DSMB). The criterion for statistical significance at the interim analyses will be set at $p<0.001$ as recommended by Peto et al (Peto R, et al. Brit J Cancer 1976;34:585.). This analysis will be conducted at arm's length from the principal investigator and the research personnel, and assess study safety/benefit based on reported participant outcomes. This interim analysis will be used to determine if the study should proceed or be stopped. The study will be stopped or continued based on the recommendation of the DSMB. If the recommendation is to stop the trial, the data will be unblinded to the investigator and the DSMB will explain their rationale for recommending that the trial be stopped. If the recommendation is to continue; the investigators will remain blinded to the interim results. If the study is stopped, all participants will be immediately notified by letter indicating the reason for the study's cessation and be directed to the study principal investigator for further questions.

DISSEMINATION OF OUTCOMES

Upon completion of the study, all participants will receive a debriefing letter outlining the study results and implications for their chronic disease care. Participants will be invited to request any future publications resulting from the study. Additional knowledge dissemination will be conducted under the umbrella of the British Columbia Alliance on Telehealth Policy and Research (www.BCATPR.ca) led by Dr. Lear. The BCATPR has developed an extensive knowledge translation process which includes bimonthly team teleconferences, the BCATPR's bimonthly newsletter (distributed to > 400 decision-makers, academics and health professionals), website (www.bcatpr.ca), creation of briefing reports, media releases and an annual workshop targeted to decision-makers, academics and health professionals. These media will be used to inform target end-users of our ongoing progress (i.e. study initiation and recruitment) and end of grant results. Upon completion, we will also conduct presentations to the participating health communities, health authorities, the Ministry of Health Services, and academics through national and international conferences as well as publication in peer-reviewed publications targeted to the health services and policy sectors (such as cardiology, general practice, nursing, rural health, telehealth and gerontology). There are also a number of patient advocacy groups and NGOs (i.e. Heart and Stroke Foundation) that we will also engage with and share the results.

IMPACT AND FUTURE DIRECTIONS

Results of this study will indicate whether the iCDM is an effective intervention to remotely facilitate patient self-management and monitoring of patient symptoms, therefore, increasing the accessibility of care for this growing patient population. If successful, the iCDM will support both rural patients and their PCP in the management of their conditions resulting in reduced hospitalizations, improved quality of life and patient/provider satisfaction. This will help to reduce the treatment gap in care in rural areas and robustly illustrate the benefit of telehealth for chronic disease management. We anticipate results of this study will be relevant to other areas in Canada and in urban areas as well. We will work with our health authority partners and with the Ministry of Health to implement and promote this service to ensure that the translation from research to practice occurs in a timely manner. Based on the outcomes of this investigation, conduct future research will entail: 1) study the iCDM in urban populations as an adjunct to current hospital-based ambulatory care, 2) investigate additional technology such as mobile phone integration and voice-activated data entry, 3) investigate additional chronic diseases such as cancer and HIV, and 4) conduct a multi-centred trial across Canada.

APPENDICES
iCDM Overview

The Internet-based chronic disease management program (iCDM) will be guided by the BC Expanded Chronic Care Model using the principles of patient self-management and integrated care (between and within providers). The iCDM has been designed for patients with one or more of the following: ischemic heart disease, heart failure, diabetes, chronic kidney disease and COPD. We will use a simple user interface appropriate for our target patients (i.e. large font, audio prompts, easy navigation, etc.). The iCDM will have four interfaces described below: 1) the patient interface; 2) the nurse interface (including allied health professionals); 3) the primary care provider (PCP) interface; 4) the administrative interface. A modest electronic health record (EHR) will include patient demographics, medical history, medications, lab data and other data entered into the iCDM by the patient and/or nurse. Patients, the iCDM nurse and the patient's PCP will have access to the EHR. The iCDM will be developed using the information systems standards as outlined by the Canada Health Infoway Standards Collaborative (http://www.infoway-inforoute.ca/lang-en/standards-collaborative).

The iCDM will support patient self-management through collaborative planning and goal setting, education and skill development, support for behaviour change, and regular patient monitoring with follow-up. In addition, it will work to support the relationship between the patient and PCP. The iCDM has been developed under the guidance of a clinical advisory committee (Table 1). The iCDM will be based on a flexible and interactive program such that patients will only be queried for their clinical data and directed to the web pages relevant to their disease conditions. For each chronic condition, we have outlined sample patient signs and symptoms to be monitored, frequency of patient provider contact and frequency of patient prompt questions on their condition (Table 2). The main premise of the iCDM is that only those patients who generate 'alerts' (see below) will be contacted by the iCDM nurse (Figure 1) allowing for the potential to manage more patients than through traditional means of required patient follow-up regardless of patient condition (see Figures 2-7 for screen shots). Across these five diseases are the following cross-cutting features: nutrition therapy, exercise therapy, psychological support, medication adherence and smoking cessation.

Patient Interface: While each disease will have its own management protocol and action plans, the general patient navigation through the iCDM will be consistent for all conditions. After logging in, patients will be directed to their data entry page to answer symptom questions and enter physical data as appropriate (i.e. weight, blood pressure and/or blood sugar). The questions are initially phrased to prompt yes/no answers (Table 2). If the patient answers 'yes' to any question, the patient will be prompted to answer how their symptoms have changed with the following possible answers (much worse, a little worse, no change, a little better, much better). Patients will also be able to enter comments in a free-text box. The data entry page will request only information relevant for their disease conditions.

Based on the patient's data entered, there are two possible scenarios: 1) the data entered is within desirable limits and the patient is directed to the Main Navigation Page, or 2) an 'alert' is generated. Alerts are generated under the following four conditions: i) the patient's answers to the symptoms questions indicate they are unwell or their symptoms are progressing, ii) the patient's entered data is outside of pre-set limits, iii) the patient enters a comment in the free text comment box below their data entry page, and iv) the patient does not enter data for a pre-specified period of time. Once an alert is generated, the patient receives a message indicating that the iCDM nurse will contact them within the next 24 hours during regular business hours (ie: not during the weekend, holidays or after hours). This message will indicate that if they are feeling unwell they should seek medical attention. If the alert is generated on a Friday, weekend or holiday, the patient will receive a message indicating the nurse will contact them on the next business day and indicate if they feel unwell to seek medical attention. This will be emphasized to the patient at the time of consent. An email will be immediately sent to the iCDM nurse indicating that an alert has been generated. The alert will also be placed into the nurse's inbox in the iCDM. The nurse will respond to alerts by contacting the patient by telephone (see below).

In addition to the symptom report questions, patients will be prompted every eight weeks to answer questions regarding their diet, physical activity habits, experience with stress/anxiety/depression, medication adherence and smoking status (if applicable, Table 3). Special alerts will be generated if the patient's responses to these questions indicate they would benefit from addition support/counselling. If alerts are generated for either diet or physical activity, patients will be prompted by the question: "Based on your responses, you may benefit from further support from a <insert dietitian and/or exercise specialist>. Would you like the <insert dietitian and/or exercise specialist> to contact you to discuss your <diet and/or physical activity?" If the patient answers 'yes' they will be contacted by the appropriate health professional who will counsel the patient in this area and provide

an appropriate diet or exercise ‘prescription’. The dietitian has expertise in dietary management of patients with chronic disease through experience working in various chronic disease management outpatient clinics at St. Paul’s Hospital. Exercise will be prescribed as need by an American College of Sports Medicine Exercise Specialist (C) who has experience in prescribing both supervised and home exercise programs in patients with a variety of chronic diseases. The intensity of the exercise will be prescribed in accordance to the patient symptoms and disease condition in order to realize the benefits of improved physical activity/exercise while minimizing patient risk. If an alert is generated for either stress/anxiety/depression, medication adherence and smoking status (if applicable), this alert will be directed to the iCDM nurse to follow-up by telephone and counsel the patient appropriately. In the case of stress/anxiety/depression this may result in the patient being recommended to see their family physician/nurse practitioner or direct to specific psychological supports in the patient’s community.

Following patient data entry, and any alert messages, the patient will be directed to his/her personal Main Navigation Page where s/he can then access the following:

- Progress Page: Feedback is a key component to reinforce self-management skills. The Progress Page will allow patients to view trends of their data over time integrated with their reported symptoms and any alerts that have been generated. This page will be designed so that patients can see the link between their physical measures (signs) and well-being (symptoms) and learn to identify at what point medical attention is necessary to avoid an acute admission. In addition, they will be able to view, and revise, their current goals (outlined by the patient upon beginning the iCDM) as well as view any feedback by the iCDM nurse.
- Action Plans: Where present, we will use action plans already in place for the patient and include them into the iCDM. If no action plans exist for the patient, they will be developed in partnership between the patient, the iCDM nurse and other providers involved with the patient’s care.
- Public Forum: Throughout the participation in the iCDM, patients will have the opportunity to participate in the public forum by posting comments for other iCDM participants to view and comment on. These forums will allow for a sense of community among the iCDM participants and will be monitored/reviewed by the iCDM nurse for accuracy.
- Education: Patients will have access to disease-specific education that will be adapted from curriculum currently in use in the province’s ambulatory care clinics.
- Additional Resources/Links to external websites.
- Patient Administration: Patients will be able to change their password, update their profile and enter a vacation period when they will be unable to enter regular data.

Nurse Interface: After logging into the iCDM, nurses will be directed to their alert inbox. This will consist of a list of patients who have generated alerts. The alert will include the patient’s name, brief reason for the alert, and date and time of the alert. The list of alerts will be much like an email inbox with unread alerts in bold font and alerts that have been read in regular font. By clicking on the alert, the nurse can view the specifics of the alert and the patient’s progress page, and begin to resolve the alert. The nurse will not be able to delete the alert; alerts will only be removed once they have been resolved. The nurse will then contact the patient by phone to discuss the alert generated and result in one or more of the following outcomes:

1) provide general counselling which may include titration of medications;
2) referral to an iCDM allied health professional (dietitian, exercise specialist);
3) direct the patient to their PCP, and send a report (via email or telefax) to the patient’s physician/nurse practitioner;
4) direct the patient to the nearest emergency room.

The nurse will document the action, enter feedback into the patient’s progress page and resolve the alert. The nurse will also have access to a list of all active patients, their EHR and the patient Main Navigation Page which will give them access to the same features as the patient.

Primary Care Provider (PCP) Interface: After logging in, the patient’s PCP will have access to a list of patients registered in the iCDM under their care. From that list, the physician/nurse practitioner can select a patient and go to the patient Main Navigation Page which will give them access to the same features as the patient. In addition, the physician/nurse practitioner will have access to any reports on that patient sent by the iCDM nurse, an area for physician/nurse practitioner notes and the ability to provide feedback to the patient on their progress page.

Administrator Interface: After logging in, the administrator will be directed to the main administration page where they will be able to register new patients and their PCP, and any new iCDM nurse and allied health professionals. The administrator will also be able to edit the patient Resources/Links and Education sections for content. The administrator will not be able to view any patient information other than that required for registering the patient into the iCDM.

Table1: Clinical Advisory Committee

Name	Health Authority	Profession	Practice Area
Dan Bilkser	Vancouver Coastal Health	Psychologist	Chronic diseases, anxiety, stress, depression
Jane Burns	PHC	Nurse	Respiratory
Marna DeSousa	Northern Health	Nurse	Chronic diseases
Sabrina Gill	PHC	Physician	Endocrinology
Dan Horvat	Northern Health	Physician	Primary care
Andrew Ignaszewski	PHC	Physician	Cardiology
Annemarie Kaan	PHC	Nurse	Cardiology
Adera Levin	PHC/PHSA	Physician	Nephrology
Karen Mahoney	Fraser Health	Nurse	Nephrology
Ray Markham	Northern Health	Physician	Primary care
John Pawlovich	Northern Health	Physician	Primary care
Stefan vanEeden	PHC	Physician	Respiratory
Melodie Yong	PHC	Dietitian	Cardiology, endocrinology, nephrology
Minetaro Naruki-van Velzen	PHC	Exercise specialist	Cardiology
Patient 1	Name withheld for confidentiality reasons.		
Patient 2	Name withheld for confidentiality reasons.		

PHC- Providence Health Care
PHSA- Provincial Health Services Authority

Table 2: Finalized patient symptom questions and physical data for the patient to enter into the iCDM.

**Ischemic Heart Disease**	**Heart Failure**	**Diabetes**	**Chronic Kidney Disease**	**COPD**
On exertion do you experience any discomfort, pain, pressure or tightness in you chest, throat or arm?<br><br>During rest, do you experience any discomfort, pain, pressure or tightness in you chest, throat or arm?<br><br>Have you felt your heart racing, fluttering or missing beats more than normal?<br><br>Do you have feelings of dizziness or light-headedness?	Do you feel your breathing is more difficult?<br><br>Are your ankles more swollen, or do you feel bloated?<br><br>Did you wake up feeling more short of breath?<br><br>Have you felt your heart racing, fluttering or missing beats more than normal?<br><br>Do you have feelings of dizziness or light-headedness?<br><br>Do you have less energy than usual?	Do you have feelings of dizziness or light-headedness?<br><br>Do you have any symptoms of feeling thirsty, frequent urination, fatigue or blurred vision?<br><br>Do you have any feelings of numbness, sweating, shaking or confusion?<br><br>Do you have any feelings of being unwell, fever, nausea?	Have you noticed a decrease in your appetite?<br><br>Are you experiencing nausea?<br><br>Compared to last week, do you have less energy?<br><br>Have you noticed foam in your urine?<br><br>Do you have a metallic taste in your mouth?<br><br>Are your ankles more swollen, or do you feel bloated?<br><br>Do you have less energy than usual?<br><br>Do you have any symptoms of feeling thirsty, frequent urination, fatigue or blurred vision?	Are you coughing more than usual?<br><br>Are you producing more sputum than usual?<br><br>Has your sputum changed colour to yellow, green or rust?<br><br>Has your sputum changed colour to yellow, green or rust?<br><br>Do you feel your breathing is more difficult?
Weight<br>Blood pressure	Weight	Weight<br>Blood pressure<br>Blood sugar	Weight<br>Blood pressure<br>*Note: for patients using dialysis, heart failure questions will be used as well.*	N/A

Table 3: Patient prompted questions for diet, physical activity habits, experience with stress/anxiety/depression, medication adherence and smoking status to be answered every eight weeks.

Smoking

1. Do you currently use any tobacco products, or have recently quit using tobacco products in the past 6 months?

❑ No ❑ Yes (generates alert)

Medication Adherence

1. Are you able to take your medications at the correct dose and time more than 80% of the time?

❑ No (generates alert) ❑ Yes

Diet and Nutrition

All are yes/no type answers.

1. I eat at least 4 servings of vegetables a day.
2. I eat at least 3 servings of fruit a day.
3. I eat nuts and/or soy protein at least 4 times per week.
4. I eat red meat (beef, pork, lamb, processed/luncheon meat) more than once per week.
5. I eat white meat (fish, chicken) more than 3 times per week.
6. I eat vegetable sources of protein (tofu, legumes, nuts, etc.) more than meat.
7. I eat whole grain, whole wheat and high fibre foods most of the time.
8. I use hard margarine and or shortening in my foods.
9. I eat packaged baked goods and processed foods.
10. I use vegetable oils like canola, olive or safflower in my cooking.
11. I drink low fat dairy (skim milk or 1% milk).
12. I eat cheese more than 3 times per week.
13. I drink less than 2 drinks of alcohol a day (glass of wine, bottle of beer, shot of liquor). <for women>
13. I drink less than 3 drinks of alcohol a day (glass of wine, bottle of beer, shot of liquor). <for men>

The alert is based on a total score of these 13 questions. For the questions in green, an answer of 'yes' is scored as a 1, for the questions not highlighted, an answer of 'no' is scored a 1. An alert is generated if the score is <9.

Physical Activity

1. How many times a week do you usually do 20 minutes or more of vigorous physical activity that makes you sweat or puff or pant? (for example, jogging, heavy lifting, digging, aerobics or fast bicycling)

❑ greater than or equal to 3 times per week	score: 4
❑ 1-2 times per week	2
❑ None	0

2. How many times a week do you usually do 30 minutes or more of moderate physical activity or walking that increases your heart rate or makes you breath harder than normal? (for example, mowing the lawn, carrying light loads, bicycling at a regular pace or playing doubles tennis)

❑ greater than or equal to 5 times per week	score: 4
❑ 3-4 times per week	2
❑ 1-2 times per week	1
❑ None	0

Total score question 1 and question 2. If score 3 or less, alert generated to exercise specialist.

Mood/depression

Alerts generated when patient answers four or more to any question.

Over the last two weeks, how much were you bothered by:
1. Feeling stressed?

0	1	2	3	4	5	6	7	8	9
Not at all			a little			moderately			severely

2. Feeling sad, down or uninterested in life?

0	1	2	3	4	5	6	7	8	9
Not at all			a little			moderately			severely

3. Feeling anxious or nervous?

0	1	2	3	4	5	6	7	8	9
Not at all			a little			moderately			severely

4. Feeling angry?

0	1	2	3	4	5	6	7	8	9
Not at all			a little			moderately			severely

5. No having the social support you feel you need?

0	1	2	3	4	5	6	7	8	9
Not at all			a little			moderately			severely

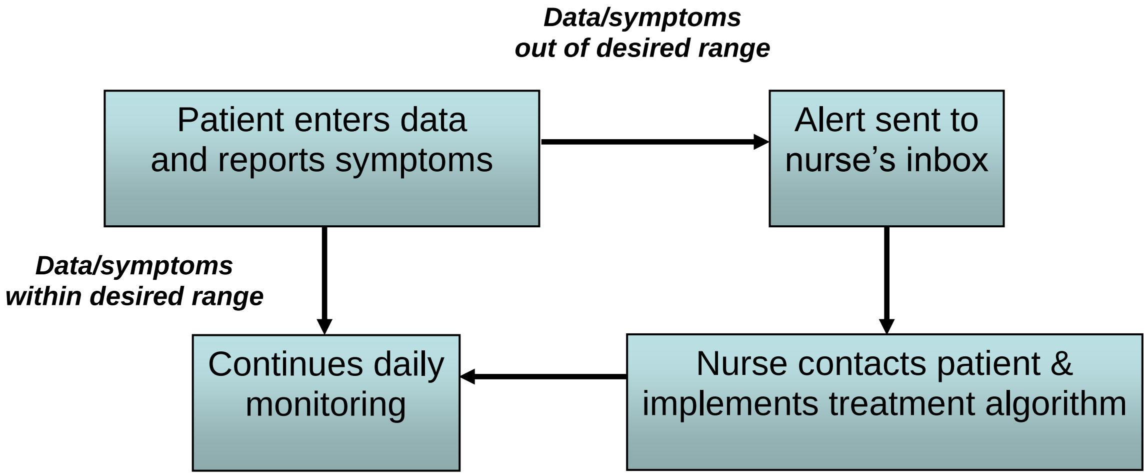

Figure 1: Schematic premise of the patient data entry, alert generation and resolution.

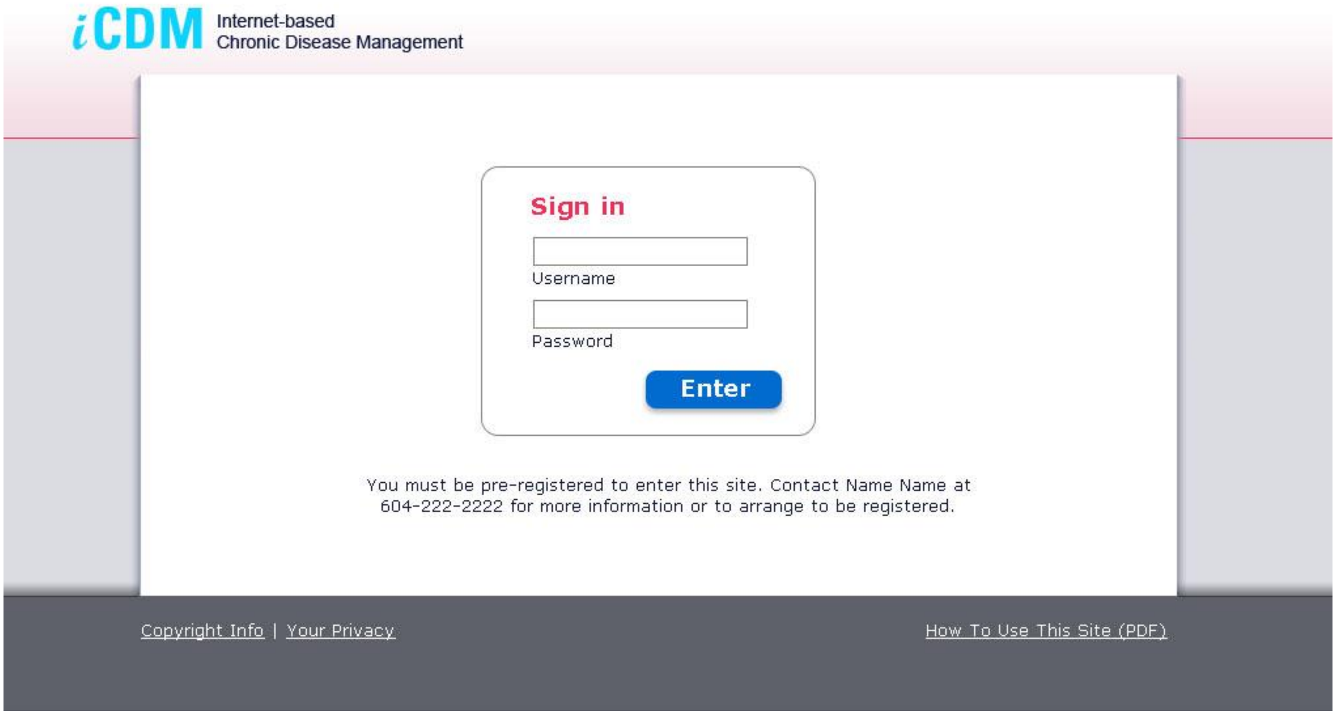

Figure 2: Patient log-in.

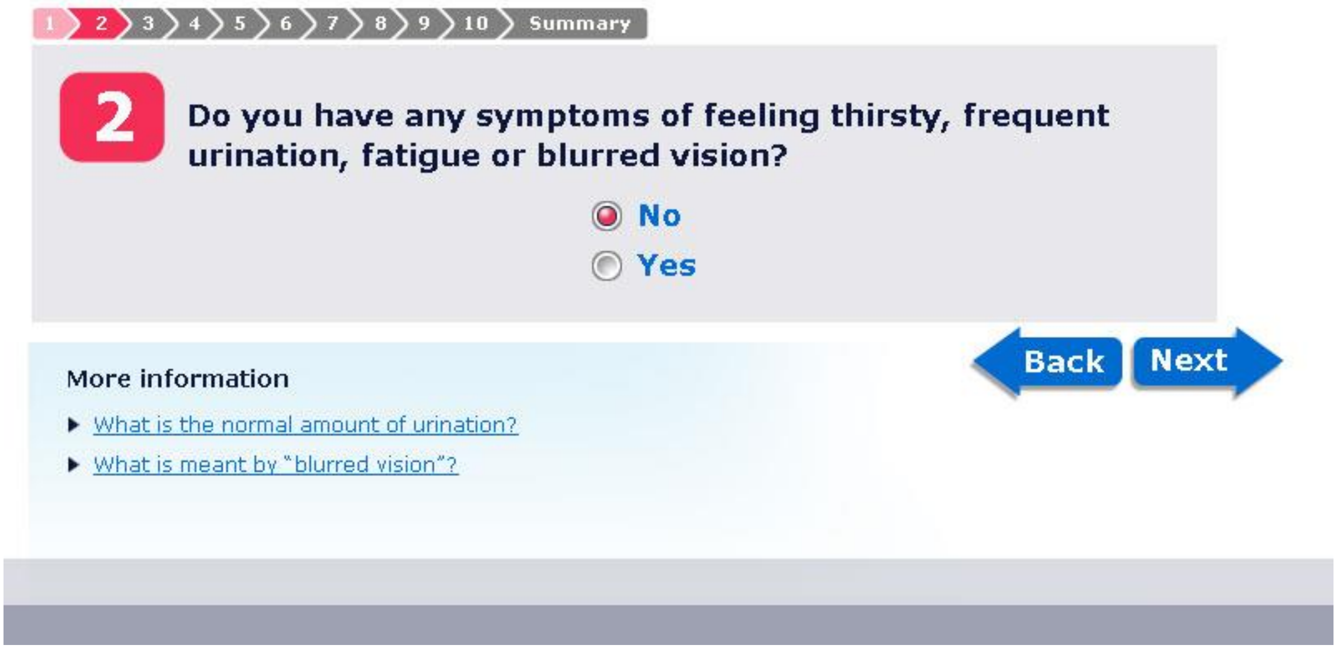

Figure 3: Layout of symptom questions. If the patient answers 'yes' to any question, the patient will be prompted to answer how their symptoms have changed with the following possible answers (much worse, a little worse, no change, a little better, much better).

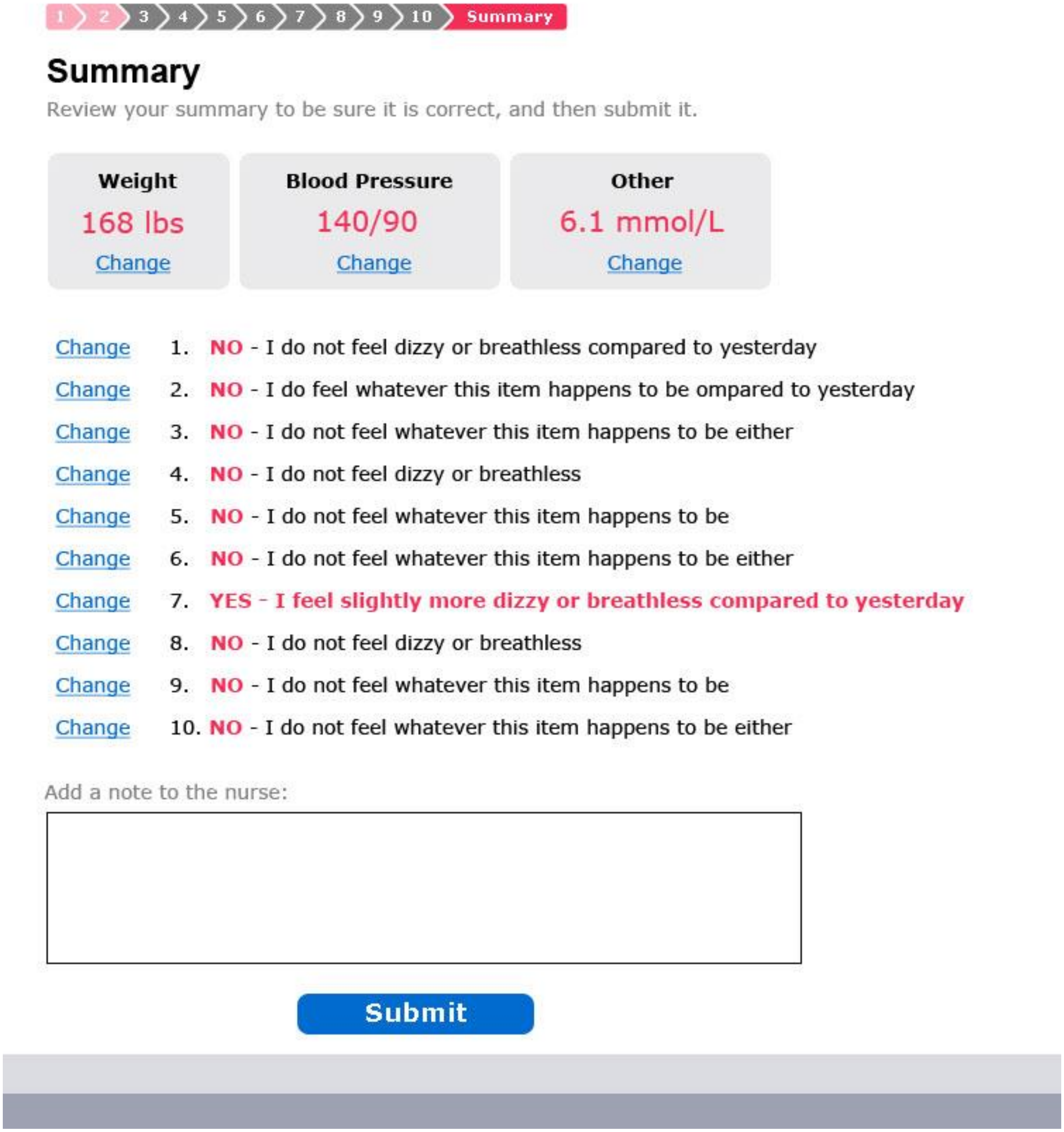

Figure 4: Sample summary of completed patient symptom report.

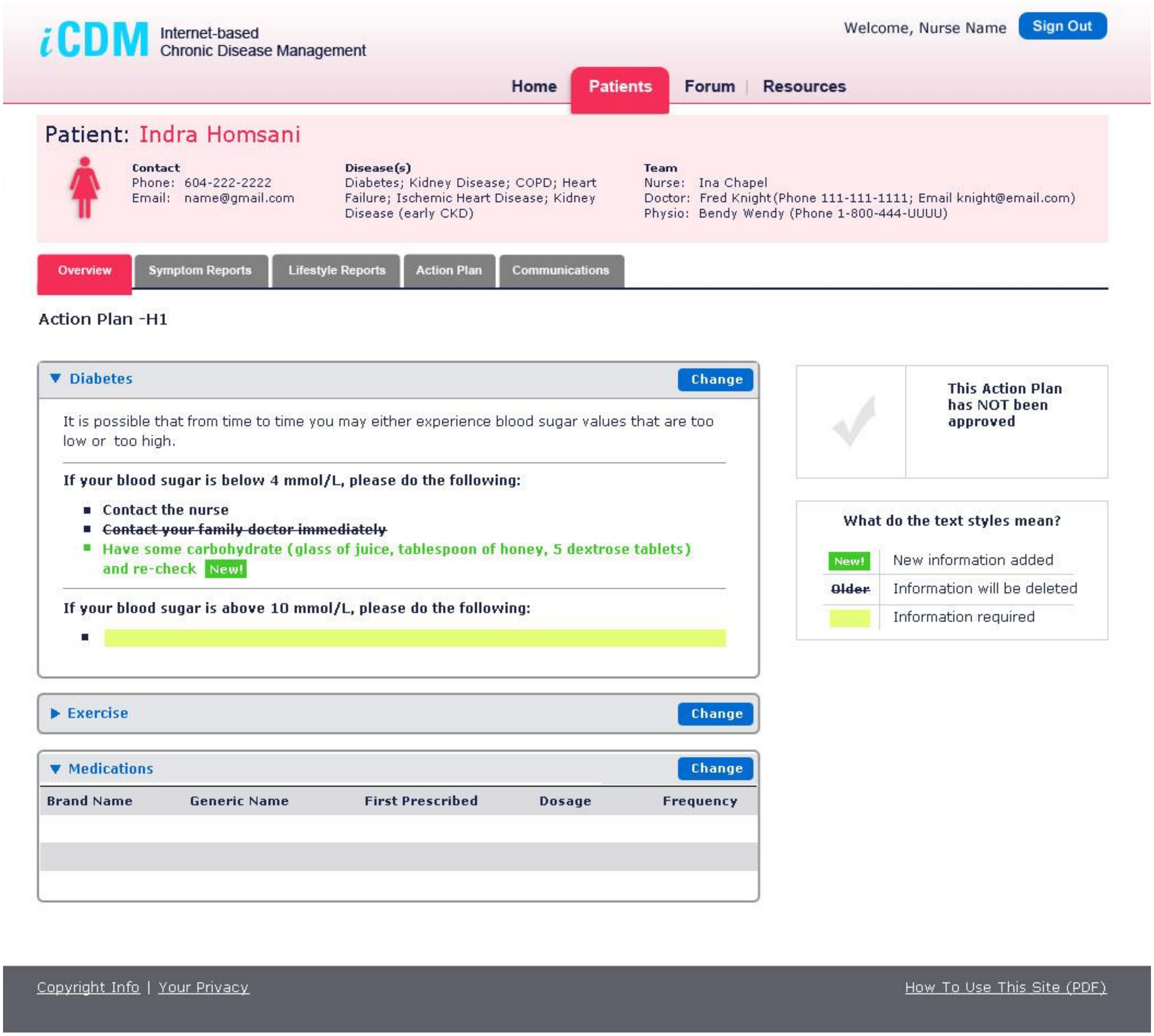

Figure 5: Sample patient action plan- nurse's view.

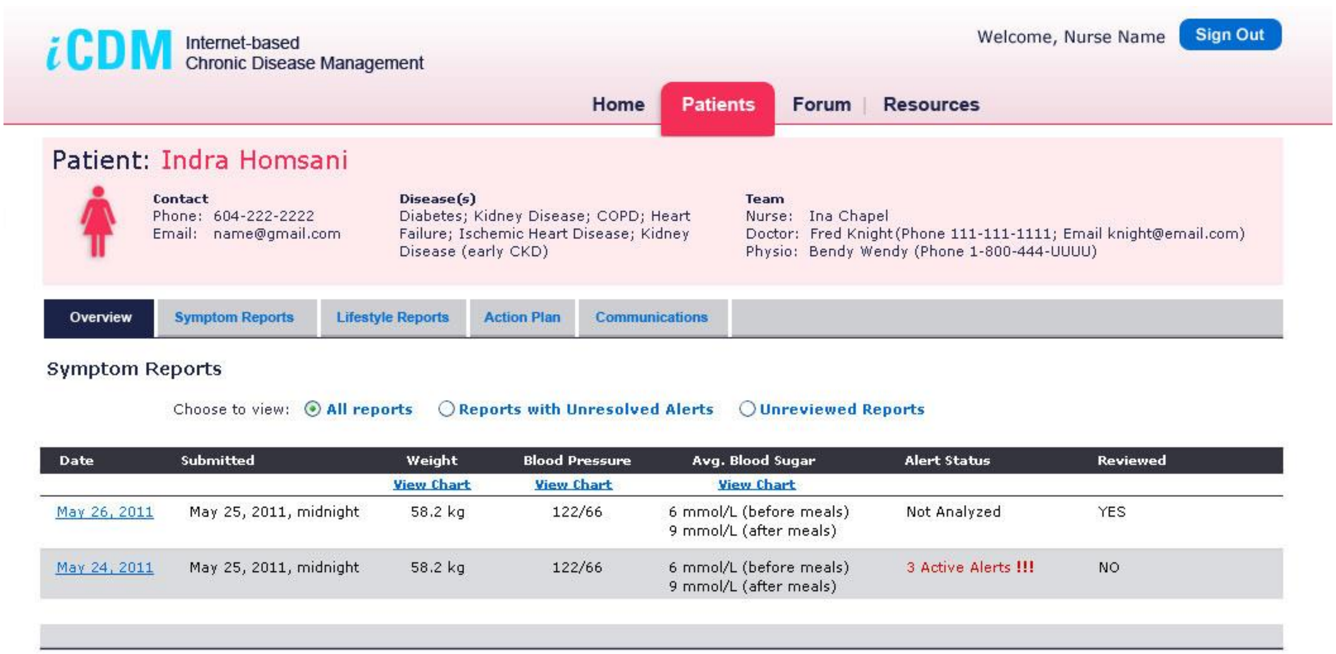

Figure 6: Patient summary page- nurse’s view.

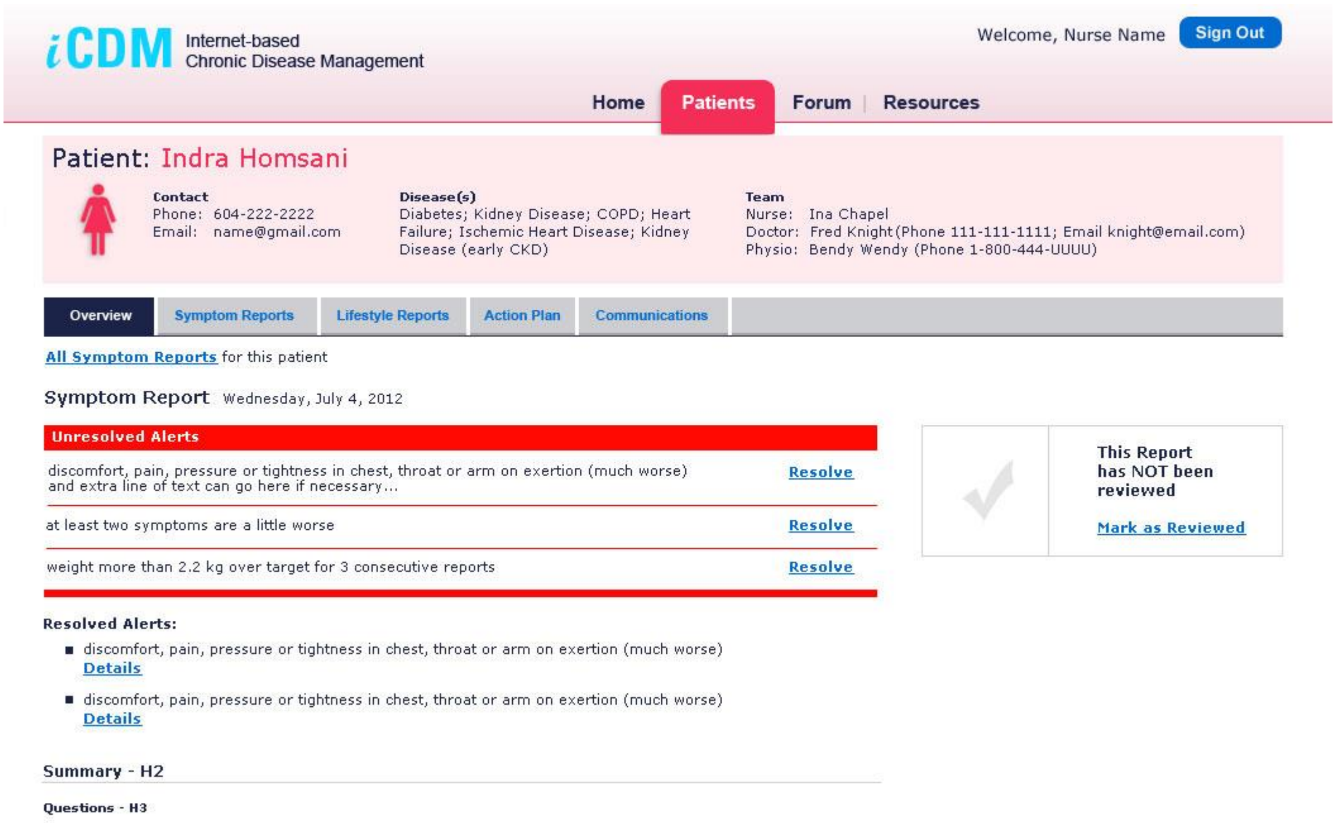

Figure 7: Patient alert summary- nurse’s view.

REFERENCES

1. Broemeling AM, Watson DE, Prebtani F. Population patterns of chronic health conditions, co-morbidity and healthcare use in Canada: implications for policy and practice. Healthc Q. 2008;11:70-6.
2. Organization WH. Facing the facts: The impact of chronic disease in Canada. Geneva: World Health Organization2005.
3. Broemeling AM, Watson D, Black C. Chronic conditions and co-morbidity among residentsof British Columbia. Vancouver, BC: Centre for Health Services and Policy Research2005 February 2005.
4. DesMeules M, Pong R. How Healthy are Rural Canadians: An Assessment of their Health Status and Health Determinants. Ottawa: Canadian Insitute for Health Information2006.
5. Hollander MJ, Kadlec H, Hamdi R, Tessaro A. Increasing value for money in the Canadian healthcare system: New findings on the contribution of primary care services. Healthcare Quart. 2009;12:30-42.
6. Sarnak MJ, Levey AS, Schoolwerth AC, Coresh J, Culleton B, Hamm LL, McCullough PA, Kasiske BL, Kelepouris E, Klag MJ, Parfrey P, Pfeffer M, Raij L, Spinosa DJ, Wilson PW. Kidney disease as a risk factor for development of cardiovascular disease: a statement from the American Heart Association Councils on Kidney in Cardiovascular Disease, High Blood Pressure Research, Clinical Cardiology, and Epidemiology and Prevention. Circulation. 2003;108:2154-69.
7. Meystre S. The current state of telemonitoring: a comment on the literature. Telemed J E Health. 2005;11:63-9.
8. Neubeck L, Redfern J, Fernandez R, Briffa T, Bauman A, Freedman SB. Telehealth interventions for the secondary prevention of coronary heart disease: a systematic review. Eur J Cardiovasc Prev Rehabil. 2009;16:281-9.
9. Dellifraine JL, Dansky KH. Home-based telehealth: a review and meta-analysis. J Telemed Telecare. 2008;14:62-6.
10. Pare G, Jaana M, Sicotte C. Systematic review of home telemonitoring for chronic diseases: the evidence base. J Am Med Inform Assoc. 2007;14:269-77.
11. Clark RA, Inglis SC, McAlister FA, Cleland JG, Stewart S. Telemonitoring or structured telephone support programmes for patients with chronic heart failure: systematic review and meta-analysis. Bmj. 2007;334:942.
12. Jaana M, Pare G, Sicotte C. Home telemonitoring for respiratory conditions: a systematic review. Am J Manag Care. 2009;15:313-20.
13. Highway OoHatI. Telehomecare: An Overview - Background Paper for Discussion. Ottawa: Policy and Consultation Branch, Health Canada1998.
14. Romanow R. Building on Values: The Future of Health Care in Canada. Ottawa: Health Canada2002.
15. Association CHC. Integration through Information Technology for Home Care in Canada.2008.
16. Garcia-Lizana F, Sarria-Santamera A. New technologies for chronic disease management and control: a systematic review. J Telemed Telecare. 2007;13:62-8.
17. Barlow J, Singh D, Bayer S, Curry R. A systematic review of the benefits of home telecare for frail elderly people and those with long-term conditions. J Telemed Telecare. 2007;13:172-9.
18. Hersh WR, Hickam DH, Severance SM, Dana TL, Pyle Krages K, Helfand M. Diagnosis, access and outcomes: Update of a systematic review of telemedicine services. J Telemed Telecare. 2006;12 Suppl 2:S3-31.
19. Bensink M, Hailey D, Wotton R. A systematic review of successes and failures in home telehealth: Preliminary results. J Telemed Telecare. 2006;12:8-16.
20. Maric B, Kaan A, Ignaszewski A, Lear SA. A systematic review of telemonitoring technologies in heart failure. Eur J Heart Fail. 2009;11:506-17.

21. Riegel B, Moser DK, Glaser D, Carlson B, Deaton C, Armola R, Sethares K, Shively M, Evangelista L, Albert N. The Minnesota Living With Heart Failure Questionnaire: sensitivity to differences and responsiveness to intervention intensity in a clinical population. Nurs Res. 2002;51:209-18.
22. Wu RC, Delgado D, Costigan J, Maciver J, Ross H. Pilot study of an Internet patient-physician communication tool for heart failure disease management. J Med Internet Res. 2005;7:e8.
23. LaFramboise LM, Woster J, Yager A, Yates BC. A technological life buoy: patient perceptions of the Health Buddy. J Cardiovasc Nurs. 2009;24:216-24.
24. Delgado DH, Costigan J, Wu R, Ross HJ. An interactive Internet site for the management of patients with congestive heart failure. Can J Cardiol. 2003;19:1381-5.
25. Goldberg LR, Piette JD, Walsh MN, Frank TA, Jaski BE, Smith AL, Rodriguez R, Mancini DM, Hopton LA, Orav EJ, Loh E. Randomized trial of a daily electronic home monitoring system in patients with advanced heart failure: the Weight Monitoring in Heart Failure (WHARF) trial. Am Heart J. 2003;146:705-12.
26. Westlake C, Evangelista LS, Stromberg A, Ter-Galstanyan A, Vazirani S, Dracup K. Evaluation of a Web-based education and counseling pilot program for older heart failure patients. Prog Cardiovasc Nurs. 2007;22:20-6.
27. Southard BH, Southard DR, Nuckolls J. Clinical trial of an Internet-based case management system for secondary prevention of heart disease. J Cardiopulm Rehabil. 2003;23:341-8.
28. Cavan DA, Everett J, Plougmann S, Hejlesen OK. Use of the Internet to optimize self-management of type 1 diabetes: preliminary experience with DiasNet. J Telemed Telecare. 2003;9 Suppl 1:S50-2.
29. Clarke D, Rowe I, Gribben B, Brimacombe P, Engel T. Integrated disease management pilot for diabetes. J Healthc Inf Manag. 2002;16:52-9.
30. Hebert MA, Paquin MJ, Iversen S. Predicting success: stakeholder readiness for home telecare diabetic support. J Telemed Telecare. 2002;8 Suppl 3:S3:33-6.
31. Kwon HS, Cho JH, Kim HS, Lee JH, Song BR, Oh JA, Han JH, Cha BY, Lee KW, Son HY, Kang SK, Lee WC, Yoon KH. Development of web-based diabetic patient management system using short message service (SMS). Diabetes Res Clin Pract. 2004;66 Suppl 1:S133-7.
32. Ralston JD, Revere D, Robins LS, Goldberg HI. Patients' experience with a diabetes support programme based on an interactive electronic medical record: qualitative study. Bmj. 2004;328:1159.
33. Powell J, Jennings A, Armstrong N, Sturt J, Dale J. Pilot study of a virtual diabetes clinic: satisfaction and usability. J Telemed Telecare. 2009;15:150-2.
34. Seto E, Cafazzo JA, Rizo C, Bonert M, Fong E, Chan CT. Internet use by end-stage renal disease patients. Hemodial Int. 2007;11:328-32.
35. Nguyen HQ, Carrieri-Kohlman V, Rankin SA, Slaughter R, Stulbarg MS. Pilot study of an online dyspnea self-management program for COPD. AMIA Annu Symp Proc. 2003:951.
36. Nguyen HQ, Carrieri-Kohlman V, Rankin SH, Slaughter R, Stulbarg MS. Is Internet-based support for dyspnea self-management in patients with chronic obstructive pulmonary disease possible? Results of a pilot study. Heart Lung. 2005;34:51-62.
37. Nguyen HQ, Donesky-Cuenco D, Wolpin S, Reinke LF, Benditt JO, Paul SM, Carrieri-Kohlman V. Randomized controlled trial of an internet-based versus face-to-face dyspnea self-management program for patients with chronic obstructive pulmonary disease: pilot study. J Med Internet Res. 2008;10:e9.
38. Kwon HS, Cho JH, Kim HS, Song BR, Ko SH, Lee JM, Kim SR, Chang SA, Cha BY, Lee KW, Son HY, Lee JH, Lee WC, Yoon KH. Establishment of blood glucose monitoring system using the internet. Diabetes Care. 2004;27:478-83.
39. Ralston JD, Hirsch IB, Hoath J, Mullen M, Cheadle A, Goldberg HI. Web-based collaborative care for type 2 diabetes: a pilot randomized trial. Diabetes Care. 2009;32:234-9.

40. McCarrier KP, Ralston JD, Hirsch IB, Lewis G, Martin DP, Zimmerman FJ, Goldberg HI. Web-based collaborative care for type 1 diabetes: a pilot randomized trial. Diabetes Technol Ther. 2009;11:211-7.
41. Kashem A, Droogan MT, Santamore WP, Wald JW, Bove AA. Managing heart failure care using an internet-based telemedicine system. J Card Fail. 2008;14:121-6.
42. Tura A, Quareni L, Longo D, Condoluci C, van Rijn A, Albertini G. Wireless home monitoring and health care activity management through the Internet in patients with chronic diseases. Med Inform Internet Med. 2005;30:241-53.
43. Gerber BS. The chronic disease self-management program: extending reach through the internet. Med Care. 2006;44:961-3.
44. Lorig KR, Ritter PL, Laurent DD, Plant K. Internet-based chronic disease self-management: a randomized trial. Med Care. 2006;44:964-71.
45. Whitten P, Johannessen LK, Soerensen T, Gammon D, Mackert M. A systematic review of research methodology in telemedicine studies. J Telemed Telecare. 2007;13:230-5.
46. Dorr D, Bonner LM, Cohen AN, Shoai RS, Perrin R, Chaney E, Young AS. Informatics systems to promote improved care for chronic illness: a literature review. J Am Med Inform Assoc. 2007;14:156-63.
47. Kirsch SE, Lewis FM. Using the World Wide Web in health-related intervention research. A review of controlled trials. Comput Inform Nurs. 2004;22:8-18.
48. Office T. An Environmental Scan of Clinical Applications in Telehealth in Canada. Victoria: Provincial Health Services Authority2004.
49. Information NaLCfH. An Environmental Scan of Telehealth, Electronic Health Record and Electronic Medical Record Initiatives in Newfoundland and Labrador and Canada: Office of Primary Health Care2005.
50. Kraetschmer NM, Deber RB, Dick P, Jennett P. Telehealth as gatekeeper: policy implications for geography and scope of services. Telemed J E Health. 2009;15:655-63.
51. Gustafson DH, Wyatt JC. Evaluation of ehealth systems and services. Bmj. 2004;328:1150.
52. Hailey D, Roine R, Ohinmaa A. Systematic review of evidence for the benefits of telemedicine. J Telemed Telecare. 2002;8 Suppl 1:1-30.
53. Hjelm NM. Benefits and drawbacks of telemedicine. J Telemed Telecare. 2005;11:60-70.
54. Koch S. Home telehealth--current state and future trends. Int J Med Inform. 2006;75:565-76.
55. Wootton R, Dimmick SL, Kvedar JC. Chapter 24: Conclusions. In: Wotton R, Dimmick SL, Kvedar JC, editors. Home Telehealth: Connecting care within the community: Royal Society of Medicine Press; 2006.
56. Demidenko E. Sample size and optimal design for logistic regression with binary interaction. Stat Med. 2008;27:36-46.
57. Demidenko E. Sample size determination for logistic regression revisited. Stat Med. 2007;26:3385-97.
58. Ware JE, Jr., Shelborurne CD. The MOS 36-item short-form health survey (SF-36). 1. Conceptual framework and item selection. Medical Care. 1992;30:473-83.
59. Ware JE, Jr. SF-36 health survey update. In: Maruish ME, editor. Use of psychological testing for treatment planning and outcomes assessment Vol 3 Instruments for adults: Erlbaum; 2004. p. 693-718.
60. Osborne RH, Elsworth GR, Whitfield K. The Health Education Impact Questionnaire (heiQ): an outcomes and evaluation measure for patient education and self-management interventions for people with chronic conditions. Patient Educ Couns. 2007;66:192-201.
61. Sherbourne CD, Stewart AL. The MOS social support survey. Soc Sci Med. 1991;32:705-14.
62. Herdman M, Gudex C, Lloyd A, et al. Development and preliminary testing of the new five-level version of EQ-5D (EQ-5D-5L). Qual Life Res. 2011;20:1727-36.

63. Srivastava P, Hopwood N. A practical Iterative framework for qualitative data analysis. Int J Qual Methods. 2009;8:76-84.
64. Thomas DR. A general inductive approach for analyzing qualitative evaluation data. Am J Eval. 2006;27:237-46.
65. Patton MQ. Qualitative Evaluation and Research Methods. London: Sage; 2002.
66. Norman CD, Skinner HA. eHEALS: The eHealth Literacy Scale. J Med Internet Res. 2006;8:e27.
67. Radloff LS. The CES-D scale: A self-report depression scale for research in the general population. Applied Psychological Measurement. 1977;1:385-401.
