# Supplementary material for: Assessment of an Interactive Digital Health–Based Self-management Program to Reduce Hospitalizations Among Patients With Multiple Chronic Diseases: A Randomized Clinical Trial
Source: JAMA Netw Open. 2021 Dec 28;4(12):e2140591. doi: 10.1001/jamanetworkopen.2021.40591 (PMC12243620; doi:10.1001/jamanetworkopen.2021.40591)
Supplement: Supplement 2. — eTable 1. Intervention Participant Symptom-Related Questions and Biometric Data to Enter for Each of the 5 Chronic Diseases eTable 2. Default Alert Thresholds for Biometric Data eTable 3. Baseline and Follow-up Values of Quality of Life Using the Medical Outcomes Study 36-Item Short Form Survey, Version 2 eTable 4. Baseline and Follow-up Values of Self-Management Measures Using the Health Education Impact Questionnaire eTable 5. Baseline and Follow-up Values of Social Support Using the Medical Outcomes Study Social Support Scale eReferences [file jamanetwopen-e2140591-s002.pdf]

Supplementary Online Content

Lear SA, Norena M, Banner D, et al. Assessment of an interactive digital health–based self-management program to reduce hospitalizations among patients with multiple chronic diseases: a randomized clinical trial. *JAMA Netw Open*. 2021;4(12):e2140591. doi:10.1001/jamanetworkopen.2021.40591

eTable 1. Intervention Participant Symptom-Related Questions and Biometric Data to Enter for Each of the 5 Chronic Diseases

eTable 2. Default Alert Thresholds for Biometric Data

e**Table 3.** Baseline and Follow-up Values of Quality of Life Using the Medical Outcomes Study 36-Item Short Form Survey, Version 2

eTable 4. Baseline and Follow-up Values of Self-Management Measures Using the Health Education Impact Questionnaire

eTable 5. Baseline and Follow-up Values of Social Support Using the Medical Outcomes Study Social Support Scale

eReferences

This supplementary material has been provided by the authors to give readers additional information about their work.

© 2021 Lear SA et al. *JAMA Network Open*.

eTable 1. Intervention Participant Symptom-Related Questions and Biometric Data to Enter for Each of the 5 Chronic Diseases

	Diabetes	Heart Failure	Ischemic Heart Disease	Chronic Renal Disease	Chronic Obstructive Pulmonary Disorder
Symptom-related Questions					
	Do you have feelings of dizziness or light-headedness?	Do you feel your breathing is more difficult?	On exertion do you experience any discomfort, pain, pressure or tightness in you chest, throat or arm?	Have you noticed a decrease in your appetite?	Are you coughing more than usual?
	Do you have any symptoms of feeling thirsty, frequent urination, fatigue or blurred vision?	Are your ankles more swollen, or do you feel bloated?	During rest, do you experience any discomfort, pain, pressure or tightness in you chest, throat or arm?	Do you have any symptoms of feeling thirsty, frequent urination, fatigue or blurred vision?	Are you producing more sputum than usual?
	Do you have any feelings of numbness, sweating, shaking or confusion?	Did you wake up feeling more short of breath?	Have you felt your heart racing, fluttering or missing beats more than normal?	Have you noticed foam in your urine?	Has your sputum changed colour to yellow, green or rust?
	Do you have any feelings of being unwell, fever, nausea?	Have you felt your heart racing, fluttering or missing beats more than normal?	Do you have feelings of dizziness or light-headedness?	Do you have a metallic taste in your mouth?	Do you feel your breathing is more difficult?
		Do you have feelings of dizziness or light-headedness?		Are your ankles more swollen, or do you feel bloated?	
		Do you have less energy?		Do you have less energy?	
				Are you experiencing nausea?	
Biometric Data					
	Morning blood glucose<br>Blood pressure	Weight	Weight<br>Blood pressure	Weight<br>Blood pressure	Weight

© 2021 Lear SA et al. *JAMA Network Open*.

eTable 2. Default Alert Thresholds for Biometric Data

	Alerted Target
Weight Changes (HF, CRD)	↑ 2.2 kg over 3 consecutive entries, or<br>↑ 1 kg in 2 days or 2.5 kg in 7 days (or entries), or<br>↓ 1 kg in 2 days or 2.5 kg in 7 days (or entries)
Weight Changes (diabetes)	↑ 2.2 kg over 3 consecutive entries
Blood Pressure (HF, IHD)	≥140/90 mmHg
Blood Pressure (diabetes, CRD)	≥130/80 mmHg
Blood Glucose (diabetes)	Blood glucose > 7 mmol/L before a meal<br>Blood glucose > 10 mmol/L after a meal<br>Blood glucose < 4 mmol/L anytime

HF = heart failure, IHD = ischemic heart disease, CRD = chronic renal disease

© 2021 Lear SA et al. *JAMA Network Open*.

eTable 3. Baseline and Follow-up Values of Quality of Life Using the Medical Outcomes Study 36-Item Short Form Survey (SF-36), Version 2[1]

	Usual Care		iCDM		p value*
	Baseline	Follow-up	Baseline	Follow-up	
Physical functioning	55 (35,75)	50 (25,70)	65 (35,85)	62.5 (35,85)	0.05
Role functioning/physical	25 (0,75)	25 (0,100)	50 (0,100)	50 (0,100)	0.07
Role functioning/emotional	100 (33,100)	100 (0,100)	100 (33,100)	100 (67,100)	0.16
Energy/fatigue	50 (40,65)	55 (40,65)	60 (40,70)	55 (45,75)	0.21
Emotional well-being	80 (64,92)	80 (64,92)	80 (68,88)	80 (72,92)	0.08
Social functioning	75 (50,100)	75 (50,100)	87.5 (63,100)	87.5 (63,100)	0.10
Pain	52 (41,74)	52 (31,74)	62 (41,84)	62 (41,82)	0.48
General Health	52 (35,72)	52 (35,72)	62 (40,72)	62 (40,77)	0.42

Data presented as medians (25th and 75th percentiles).

* differences in the change between the groups tested using a one-tailed Wilcoxon rank-sum test.

© 2021 Lear SA et al. *JAMA Network Open*.

eTable 4. Baseline and Follow-up Values of Self-Management Measures Using the Health Education Impact Questionnaire (heiQ)[2]

	Usual Care		iCDM		p value*
	Baseline	Follow-up	Baseline	Follow-up	
Positive and Active engagement in life	4.8 (4.0,5.2)	4.8 (4.0,5.0)	5.0 (4.8,5.4)	5.0 (4.4,5.4)	0.05
Health directed behaviour	4.0 (2.5,5.0)	3.8 (2.8,5.0)	4.3 (2.9,5.1)	4.5 (3.5,5.3)	0.06
Skill and technique acquisition	4.6 (4.0,5.0)	4.6 (4.0,5.0)	4.8 (4.2,5.0)	5.0 (4.4,5.2)	<0.01
Constructive attitude shift	5.0 (4.2,5.6)	5.0 (4.6,5.4)	5.0 (4.9,5.6)	5.0 (4.8,5.8)	0.11
Self-monitoring and insight	5.0 (4.6,5.3)	5.0 (4.7,5.4)	5.0 (4.9,5.4)	5.1 (5.0,5.6)	0.02
Health service navigation	5.0 (4.8,5.8)	5.0 (5.0,5.8)	5.0 (5.0,5.8)	5.2 (5.0,6.0)	0.18
Social integration and support	4.8 (4.2,5.2)	5.0 (4.0 ,5.4)	5.0 (4.4,5.3)	5.0 (4.6,5.4)	0.02
Emotional Wellbeing	4.5 (3.7,5.3)	4.7 (3.7,5.2)	4.8 (4.0,5.5)	5.0 (4.5,5.5)	<0.01

Data presented as medians (25th and 75th percentiles).

* differences in the change between the groups tested using a one-tailed Wilcoxon rank-sum test.

© 2021 Lear SA et al. *JAMA Network Open*.

eTable 5. Baseline and Follow-up Values of Social Support Using the Medical Outcomes Study Social Support Scale[3]

	Usual Care		iCDM		p value*
	Baseline	Follow-up	Baseline	Follow-up	
Emotional/informational support	32 (25,37)	32 (25,37)	32 (24,36)	32 (28,36)	<0.01
Tangible Support	17 (13,20)	17 (13,20)	17 (15,20)	19 (16,20)	0.24
Affectionate Support	13 (11,15)	14 (11,15)	15 (12,15)	15 (12,15)	0.29
Positive social interaction	12 (9,15)	12 (10,15)	13 (11,15)	13 (11,15)	0.38
Overall Support Index	78 (62,89)	78 (66,87)	79 (66,88)	82 (69,90)	<0.01

Data presented as medians (25th and 75th percentiles).

* differences in the change between the groups tested using a one-tailed Wilcoxon rank-sum test.

© 2021 Lear SA et al. *JAMA Network Open*.

eReferences

1. Ware JE, Jr. SF-36 health survey update. In: Maruish ME, ed. *The Use of Psychological Testing for Treatment Planning and Outcome Assessment*. Vol 3. Lawrence Erlbaum Associates; 2004:693-718.

2. Osborne RH, Elsworth GR, Whitfield K. The Health Education Impact Questionnaire (heiQ): an outcomes and evaluation measure for patient education and self-management interventions for people with chronic conditions. *Patient Educ Couns*. 2007;66(2):192-201. doi:10.1016/j.pec.2006.12.002

3. Sherbourne CD, Stewart AL. The MOS social support survey. *Soc Sci Med*. 1991;32(6):705-714. doi:10.1016/0277-9536(91)90150-B

© 2021 Lear SA et al. *JAMA Network Open*.
