# Supplementary material for: Assessment of an Interactive Digital Health–Based Self-management Program to Reduce Hospitalizations Among Patients With Multiple Chronic Diseases: A Randomized Clinical Trial
Source: JAMA Netw Open. 2021 Dec 28;4(12):e2140591. doi: 10.1001/jamanetworkopen.2021.40591 (PMC12243620; doi:10.1001/jamanetworkopen.2021.40591)
Supplement: Supplement 3. — Data Sharing Statement [file jamanetwopen-e2140591-s003.pdf]

Data Sharing Statement

Lear. Assessment of an Interactive Digital Health-Based Self-management Program to Reduce Hospitalizations Among Patients With Multiple Chronic Diseases. *JAMA Netw Open*. Published December 28, 2021. doi:10.1001/jamanetworkopen.2021.40591

Data
Data available: No

Additional Information
Explanation for why data not available: We did not receive consent from the study participants to share data.
